# ANGPTL3 orchestrates hepatic fructose sensing and metabolism

**DOI:** 10.1016/j.celrep.2025.115962

**Published:** 2025-07-09

**Authors:** Meng Zhao, Karen Y. Linde-Garelli, Zeyuan Zhang, David Toomer, Saranya C. Reghupaty, John Isaiah Jimenez, Laetitia Coassolo, Lianna W. Wat, Daniel Fernandez, Katrin J. Svensson

**Affiliations:** 1 Department of Pathology, Stanford University School of Medicine, Stanford, CA 94305, USA; 2 Stanford Diabetes Research Center, Stanford University School of Medicine, Stanford, CA 94305, USA; 3 Stanford Cardiovascular Institute, Stanford University School of Medicine, CA, Stanford, USA; 4 Department of Animal Science, University of California Davis, CA, Davis, USA; 5 Department of Structural Biology, Stanford University School of Medicine, Stanford, CA, USA; 6 Department of Chemical and Systems Biology, Stanford University School of Medicine, Stanford, CA, USA; 7 Stanford Cancer Institute, Stanford University School of Medicine, Stanford, CA, USA; 8 Macromolecular Structure Knowledge Center (MSKC), Stanford University, Stanford, CA 94305, USA; 9 Stanford ChEM-H, Stanford University, Stanford, CA 94305, USA; 10 These authors contributed equally; 11 Lead contact

## Abstract

Fructose metabolism is linked to metabolic dysfunction-associated steatotic liver disease (MASLD), but the regulatory mechanisms governing fructose uptake remain poorly understood. Here, we demonstrate that MASLD livers exhibit increased uptake of fructose-derived carbons compared to healthy livers and identify that the MASLD hepatocyte secretome can increase fructose metabolism. By performing fractionation and untargeted proteomics, we uncover a role for Angiopoietin-like 3 (ANGPTL3) as a regulator of hepatic fructose metabolism, independent of its role as a lipoprotein lipase (LPL) inhibitor. Circulating ANGPTL3 levels increase in response to fructose exposure, consistent with an action as a fructose sensor. Angptl3 knockdown in the liver resulted in a significant reduction in the uptake of hepatic fructose metabolites *in vivo* and downregulation of the facilitative hepatic fructose transporter slc2a8 (GLUT8) and fructolysis enzymes. This work demonstrates the existence of extracellular control of hepatic fructose metabolism through ANGPTL3.

## In brief

In this study, Zhao et al. identify Angiopoietin-like 3 (ANGPTL3) as a regulator of hepatic fructose metabolism in metabolic dysfunction-associated steatotic liver disease (MASLD), independent of its known role as an LPL inhibitor, and suggest its potential involvement as a fructose sensor.

## Graphical Abstract

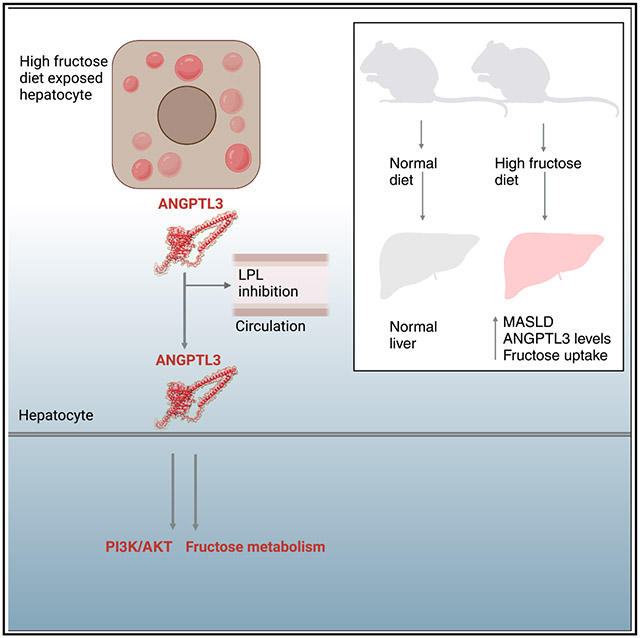

## INTRODUCTION

From 1970 to 2004, the consumption of high-fructose corn syrup increased from 0.2 kg to a remarkable 28 kg annually per person for the top 10% of the population.^1,2^ Excess fructose intake coincides with obesity and the development of metabolic dysfunction-associated steatotic liver disease (MASLD) and metabolic dysfunction-associated steatohepatitis (MASH) both in mice and humans.^3-8^ The association between fructose and liver fat is not just a consequence of high caloric input, because equicaloric glucose supplementation does not cause hepatic lipid accumulation and insulin resistance to the same extent as fructose.^9-14^ Excluding the intestine, the liver is exposed to higher fructose concentrations than other peripheral organs and is therefore disproportionally exposed to high doses of fructose.^15,16^ Once internalized, fructose is converted to fatty acids at a higher rate compared to glucose^17,18^ and also elevates *de novo* lipogenesis more robustly than glucose.^14,19^ Importantly, inhibition of the first step of fructolysis by blocking ketohexokinase (khk) effectively prevents hepatic steatosis, suggesting that inhibiting fructose metabolism is a promising treatment strategy for MASLD and MASH.^20,21^

However, the control of fructose transport remains unclear, as it is generally considered an “unregulated” process.^22,23^ The SLC2A family of hexose transporters includes several members with affinities for fructose. The facilitative hexose transporters belong to the SLC2A family (encoded by the *SLC2A1-12* genes in humans), and several of them have reported affinities for fructose, including GLUT2, GLUT5, GLUT7, GLUT8, GLUT9, and GLUT11.^24-35^ GLUT2 and GLUT5 are the main intestinal fructose transporters,^30,36^ and GLUT8 and GLUT2 are the predominant hepatic fructose transporters.^33,37^ Here, we made the interesting observation that the development of MASLD is associated with elevated hepatic uptake of fructose and fructose-derived carbons, suggesting that fructose metabolism can be controlled by external factors.

By using fractionation and unbiased proteomics of the hepatokine secretome, we identify the secreted ANGPTL3, a protein with known roles in inhibiting lipoprotein and endothelial lipases,^38^ as playing a dual role as a lipase inhibitor and an extracellular stimulator of hepatic fructose metabolism. We further found that ANGPTL3 also promotes the PI3K-AKT signaling pathway. Notably, while insulin also activates AKT,^39,40^ it does not induce fructose uptake. Antisense oligonucleotides (ASOs) targeting mouse Angptl3 have been shown to slow atherosclerosis progression, reduce hepatic lipid accumulation, and lower plasma lipid levels, including total cholesterol, low-density lipoprotein cholesterol, and triglycerides in mice.^41^ In humans, ASOs targeting ANGPTL3 in clinical trials also reduces atherogenic lipoproteins,^42^ but ANGPTL3’s role in fructose metabolism has not previously been reported.

Our work highlights the significance of identifying extracellular regulators of fructose and fructose-derived metabolite uptake, analogous to insulin-stimulated glucose uptake. This study demonstrates that ANGPTL3, in addition to controlling lipid metabolism, also plays a role in fructose metabolism, which may have consequences for the development and progression of fatty liver disease.

## RESULTS

### Hepatic uptake of fructose-derived carbons is elevated in MASLD

**Figure 1.**
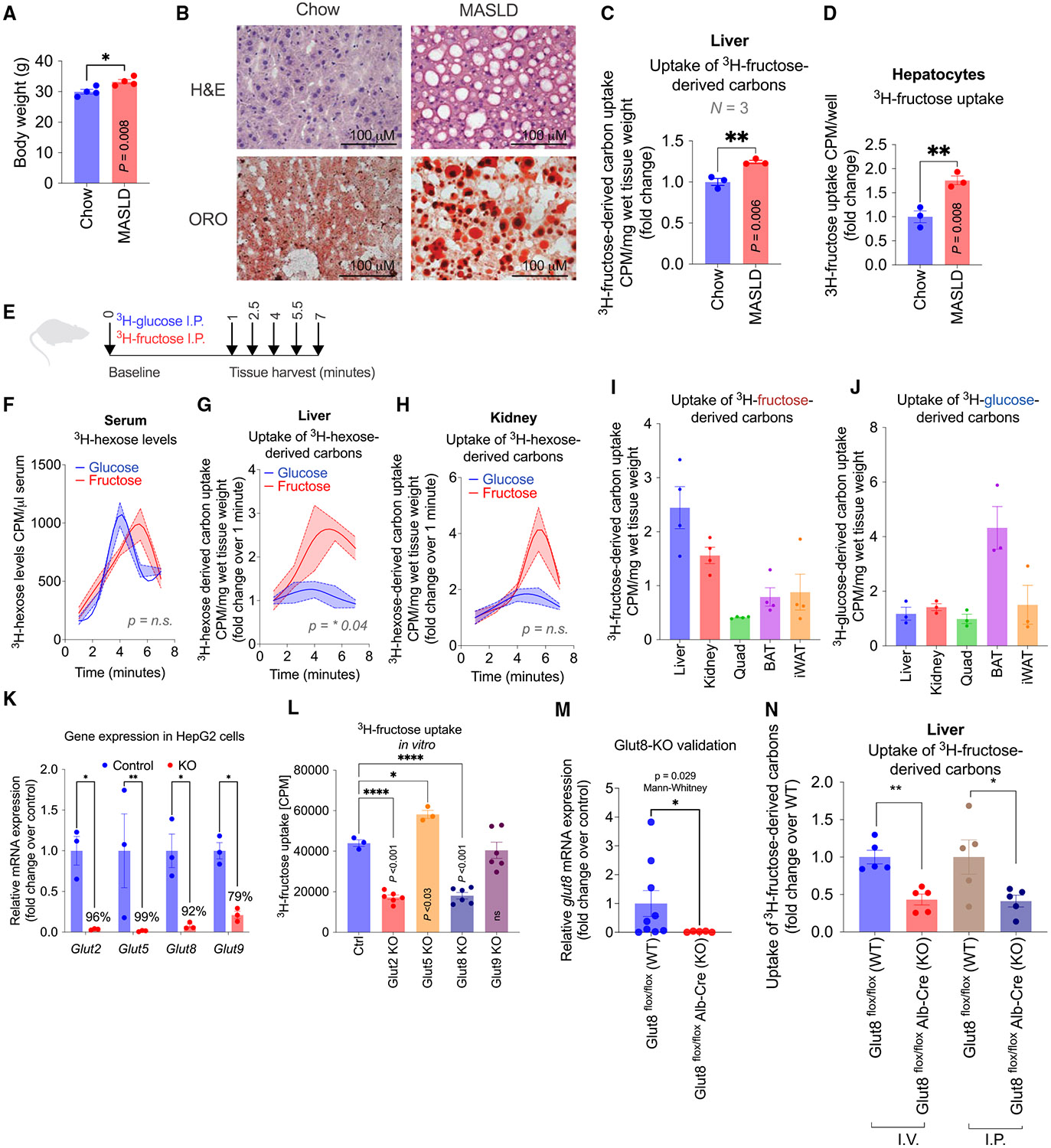
Fructose uptake is elevated in fatty livers (A) Body weight of male mice fed with chow diet or MASLD diet for 6 weeks (*N* = 4 mice per group, repeated in two independent cohorts). (B) Histological analyses using H&E and oil red O (ORO) in livers from male mice fed with chow diet or MASLD diet for 6 weeks (*N* = 5 mice per group, repeated in two independent cohorts). Scale bar: 100 μm. (C) Uptake of [3H]-fructose-derived carbons in the liver measured by [3H]-fructose incorporation via intraperitoneal injection at 100 μCi/kg in male mice fed with chow diet or MASLD diet for 6 weeks (*N* = 3 mice per group, repeated in two independent cohorts). (D) [3H]-fructose uptake measured by [3H]-fructose incorporation in primary hepatocytes isolated from male chow and MASLD mice (*N* = 3 mice per group). (E) Diagram of time-dependent *in vivo* measurements of [3H]-fructose or [3H]-glucose incorporation in male mice at 1, 2.5, 4, 5.5, and 7 min after i.p. injection. (F) Time-dependent [3H]-fructose or [3H]-glucose levels in circulation at 1, 2.5, 4, 5.5, and 7 min after i.p. injection (*N* = 3–5 male mice per group). (G) Time-dependent uptake of [3H]-fructose- or glucose-derived carbons in the liver measured by [3H]-fructose or [3H]-glucose incorporation in male mice at 1, 2.5, 4, 5.5, and 7 min after i.p. injection (*N* = 3–5 mice per group). (H) Time-dependent uptake of [3H]-fructose- or glucose-derived carbons in the kidney measured by [3H]-fructose or [3H]-glucose incorporation in male mice at 1, 2.5, 4, 5.5, and 7 min after i.p. injection (*N* = 3–5 mice per group). (I) Uptake of [3H]-fructose-derived carbons in the liver, kidney, quadriceps muscle (Quad), brown adipose tissue (BAT), and inguinal white adipose tissue (iWAT) at 2.5 min (*N* = 4 male mice per group). (J) Uptake of [3H]-glucose-derived carbons in the liver, kidney, quadriceps muscle (Quad), brown adipose tissue (BAT), and inguinal white adipose tissue (iWAT) at 2.5 min (*N* = 4 male mice per group). (K) Relative gene expression levels of *glut2*, *glut5*, *glut8*, and *glut9* in control and *glut2*-KO, *glut5*-KO, *glut8*-KO, and *glut9*-KO cell lines of HepG2 generated by CRISPR (*N* = 3 samples per group). (L) [3H]-fructose uptake measured by [3H]-fructose incorporation in control and *glut*-KO HepG2 cells (*N* = 3–6 samples per group). (M) Relative gene expression levels of *glut8* in male liver-specific *glut8*-KO (*N* = 5–9 mice per group). (N) Uptake of [3H]-glucose-derived carbons in male liver-specific *glut*8-KO mice (*N* = 5 mice per group). Data are presented as mean ± SEM. *p < 0.05, **p < 0.01, ***p < 0.001 by two-tailed Student’s t test (A, C, D, and M), one-way ANOVA (L), or two-way ANOVA (K and N).

Targeting fructose metabolism is a promising avenue for treating MASLD and MASH^19-21^; however, unlike glucose regulation, regulatory factors that control fructose uptake have not yet been identified. We observed that mice on a 6-week 20% fructose MASLD diet^4,43,44^ displayed modestly increased body weights (Figure 1A) and increased hepatic steatosis and lipid accumulation (Figure 1B). Interestingly, MASLD livers also showed elevated uptake of hepatic fructose and fructose-derived carbons compared with chow-fed mice, suggesting that fructose exposure leads to an increase in fructose metabolism (Figure 1C). Isolated primary hepatocytes from MASLD mice demonstrated a similar elevation in fructose uptake *in vitro*, suggesting that fructose exposure leads to an increase in fructose uptake by a cell-autonomous mechanism (Figure 1D). These fructose transport measurements were specific, as increasing doses of 1–10 g/L cold fructose efficiently and dose-dependently competed out 0.1, 1, and 5 μCi/mL doses of hot ^3^H-fructose (Figure S1A). When administered orally, low doses of fructose are fully metabolized by the intestine, while high fructose doses saturate the intestinal fructose metabolism machinery, leading to the delivery of fructose to the liver at concentrations up to 1 mM.^15,16,45^ Fructose is fully metabolized after 10 min after administration.^16^ To establish the temporal nature of tissue-specific uptake, ^3^H-fructose or ^3^H-2-deoxy-glucose was administered intraperitoneally (i.p.) to bypass intestinal metabolism followed by organ dissection at 0, 1, 2.5, 4, 5.5, and 7 min, to capture fructose and fructose-derived carbons into the liver (Figure 1E). To validate the use of ^3^H-fructose uptake as an indicator of hepatic fructose uptake, we measured total ^3^H accumulation in the liver following intraperitoneal administration of ^3^H-fructose. Consistent with previous studies,^45^ we observed rapid hepatic enrichment of ^3^H within 2–4 min of injection. These results confirm that our system captures early-stage uptake of fructose and its metabolites. Both ^3^H-glucose and ^3^H-fructose peaked in circulation at 4–5.5 min (Figure 1F). Compared to baseline, fructose-derived carbons were preferentially taken up by the liver at 4 min (Figure 1G), while the there was no significant difference between fructose and glucose in quadriceps skeletal muscle (Figure S1B), inguinal white adipose tissue (iWAT) (Figure S1C), brown adipose tissue (BAT) (Figure S1D), and kidney (Figure 1H). Consistent with prior studies,^45^ uptake of fructose and fructose-derived carbons at 2.5 min was significantly higher in the liver relative to other tissues (Figure 1I), while glucose-derived carbons were not significantly higher in the liver (Figure 1J). These results show that fructose and fructose-derived carbons, when compared with glucose, are preferentially taken up by the liver and elevated upon chronic fructose exposure.

### Cellular fructose uptake is facilitated by GLUT2 and GLUT8 transporters

The hexose transporter(s) are all members of the SLC2A (GLUT) family; however, GLUT2, GLUT5, and GLUT8 have been suggested to transport fructose *in vivo*. GLUT1, GLUT3, GLUT4, and GLUT6 do not transport fructose.^46,47^ GLUT2 has a high Km for glucose Km (~17 mM) and a low Km for fructose (~76 mM), GLUT5 has a high affinity for fructose, and GLUT8 and GLUT9 affinities for fructose have been reported.^33,34,46^ Two studies have reported lower fructose-induced hepatic steatosis in GLUT8 whole-body and liver-specific GLUT8 knockout (KO) mice.^48,49^ In mouse livers, *glut2* is the most highly expressed hepatic hexose transporter, followed by *glut8* and *glut9*, while *glut5* and *glut7* have no expression (Figure S1E).

To validate the measurement of cellular fructose uptake, we performed a series of competition experiments. In HepG2 cells, increasing doses of glucose compete for glucose uptake but not fructose uptake (Figure S1F); in contrast, fructose treatment competes for fructose uptake but not glucose uptake (Figure S1G), consistent with that glucose and fructose are transported through different transporters. Furthermore, we measured fructose uptake in mouse hepatocyte AML12 cells in the presence or absence of cytoB, which inhibits GLUT1-4-dependent hexose uptake. While glucose uptake was significantly inhibited with 0.1 and 1 μM cytoB in AML12 cells (Figure S1H), fructose transport was unaffected at the same doses, consistent with the presence of another fructose transporter besides GLUT2. We next used CRISPR to generate KO cell lines of the murine fructose transporters glut2, glut5, glut8, and glut9 (Figure 1K). Glut8 and glut2 ablation resulted in a 60% reduction of fructose uptake (Figure 1L). These results demonstrate that the hepatic fructose transporters in hepatocytes are glut2 and glut8, but not glut5 or glut9. Since glut2 has a low Km for fructose and given that glucose circulates at 5 mM *in vivo*, we next generated hepatocyte-specific glut8-KO mice by crossing the C57BL/6 glut8 floxed allele with C57BL/6 albumin-Cre^13,50^ (Figure S1I). To confirm the efficacy of glut8 deletion in the liver-specific glut8-KO mice, we measured *glut8* mRNA levels and observed an 80% reduction in hepatocyte levels of *glut8* compared to littermate wild-type controls (Figure 1M). Glut8-KO mice had a >50% reduction in uptake of hepatic ^3^H-fructose-derived carbons upon i.p. and intravenous (i.v.) administration, validating that Glut8 is a hepatic fructose transporter in mice (Figure 1N). These findings indicate that glut8 is a significant but not exclusive mediator of fructose transport into the liver.

### Identification of ANGPTL3 as a hepatic fructose regulator

**Figure 2.**
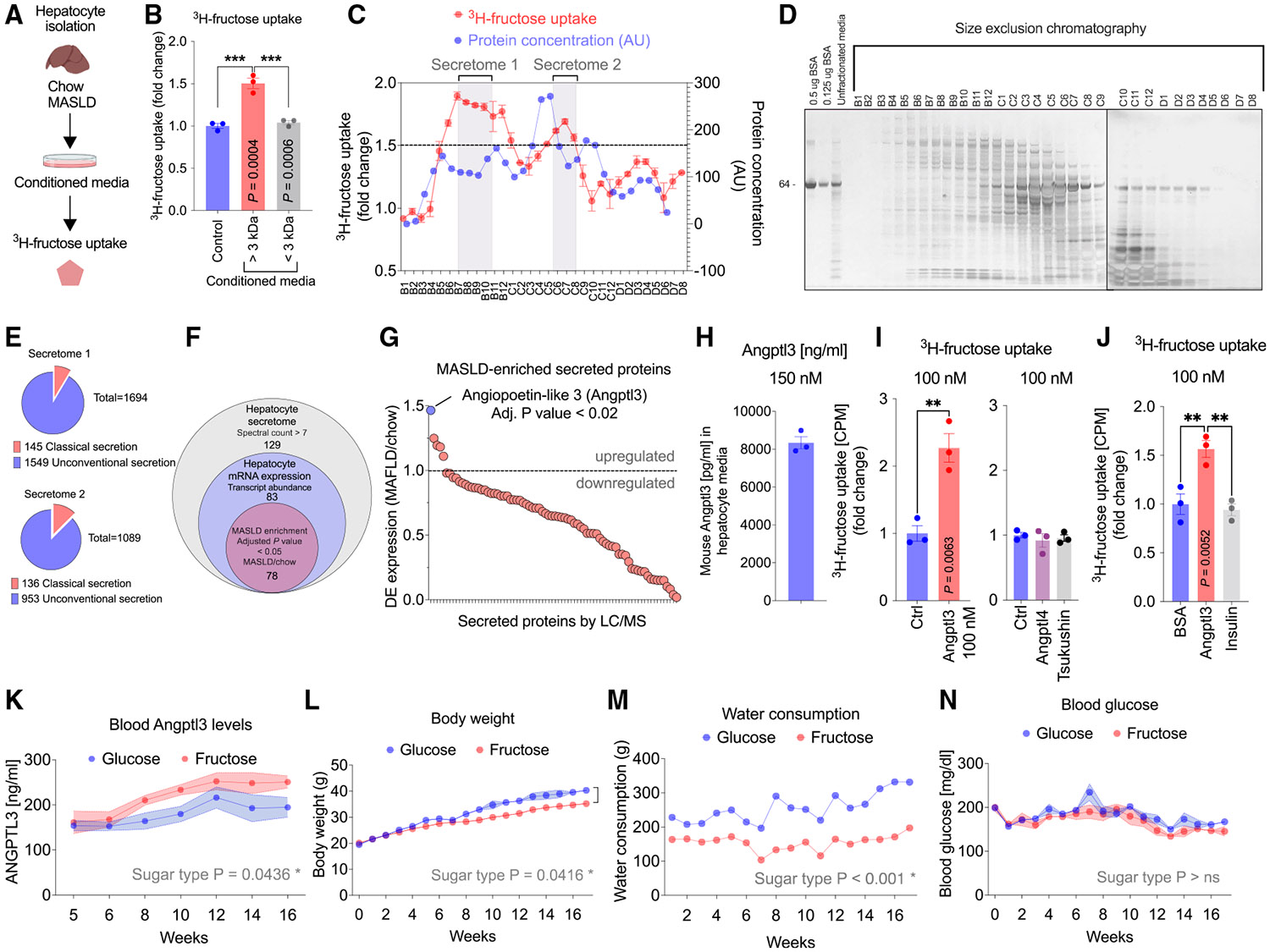
ANGPTL3 is elevated in response to dietary fructose and induces hepatocyte fructose uptake (A) Diagram of the experimental design. Male mice were fed with chow or MASLD diet for 6 weeks. Primary hepatocytes were isolated and cultured for 48 h. Conditioned media were collected, and AML12 cells were treated with conditioned media for 4 h, after which fructose uptake in AML12 cells was measured by [3H]-fructose incorporation. (B) Fructose uptake in AML12 cells treated with fractions of the conditioned medium separated by a 3K filter using >3 kDa (proteins) or <3 kDa (metabolites) (*N* = 3 samples per group, repeated in two independent experiments). (C) Fractions of MASLD media separated by fast protein liquid chromatography (FPLC) fractionation and activity in inducing fructose uptake in AML12 cells (*N* = 2 samples per fraction for the fructose uptake assays). (D) Silver stain of all fractions of MASLD media separated by FPLC fractionation. (E) Number of total proteins and secreted proteins identified by unbiased proteomics. (F) Venn diagram showing the number of proteins that overlap between datasets. (G) Fold change of differentially expressed genes in male chow and MASLD mouse livers measured by single-cell RNA sequencing. (H) Angptl3 levels in hepatocyte-conditioned media from male mice measured by ELISA (*N* = 3 samples, repeated in two independent experiments). (I) Fructose uptake measured by [3H]-fructose incorporation in AML12 cells after 24-h treatments with 100 nM recombinant mouse Angptl3 (*N* = 3 samples per group, repeated in three independent experiments), Angptl4, or Tsk (*N* = 3 samples per group). (J) Fructose uptake measured by [3H]-fructose incorporation in AML12 cells after 24-h treatments with 100 nM recombinant mouse Angptl3 or human insulin (*N* = 3 samples per group, repeated in two independent experiments). (K) Circulating male mouse Angptl3 levels measured by ELISA (*N* = 5 mice per group). (L) Weekly body weights of male mice fed fructose or glucose in drinking water (*N* = 5 mice per group). (M) Water consumption in male mice fed fructose or glucose in drinking water (*N* = 5 mice per group). (N) Weekly blood glucose of male mice fed fructose or glucose in drinking water (*N* = 5 mice per group). Data are presented as mean ± SEM. *p < 0.05, **p < 0.01, ***p < 0.001 by two-tailed Student’s t test (I), one-way ANOVA (B, I, and J), or two-way ANOVA (K, L, M, and N).

Given the evidence of a positive regulator of fructose uptake, we tested if soluble factors from MASLD hepatocytes were sufficient to facilitate fructose uptake via non-transcriptional mechanisms. When treating AML12 cells with conditioned media from chow or MASLD hepatocytes (Figure 2A), we observed a 20% significant increase by MASLD media, but no significant increase from chow hepatocytes (Figure S2A). Interestingly, the activity was only contributed by the larger >3 kDa protein fraction, but not the smaller <3 kDa fraction (Figure 2B), and the MASLD conditioned media had a distinct banding pattern (Figure S2B). We next isolated bioactive fractions by performing size exclusion chromatography using Superdex 200 on the total hepatocyte secretome (Figures 2C and 2D). The top two pooled bioactive fractions (secretome 1 and 2) were collected for untargeted mass spectrometry (Figure S2C), which identified 1,694 and 1,089 unique proteins from secretome 1 and 2, respectively (Figure 2E). To ensure sufficient coverage, we used a minimum of 7 spectral counts as a lower threshold of peptide abundance. To further refine the composition of the secretomes, we determined the fraction that contained proteins with a signal peptide (SignalP 6.0) for classical secretion,^51^ which represented 10%–20% of the conditioned media (Figure 2F). These proteins were cross-referenced to the secretome dataset with single-cell RNA sequencing of hepatocytes from chow and MASLD livers to ensure hepatocyte-specific selection, where we identified 78 proteins in common (Figure 2F). When rank-ordered based on MASLD-enriched genes, the secreted factor Angiopoietin-like 3 (Angptl3), a 460 amino acid liver-secreted protein, was the highest upregulated protein in MASLD relative to chow hepatocytes, and that was also identified by mass spectrometry in the active fractions (fold = 1.46, adjusted *p* value < 0.02) (Figures 2G and S2D; Table S1). We next generated and biochemically characterized mammalian recombinant, endotoxin-free proteins (Figure S2E) to test if Angptl3 was sufficient to induce cellular fructose uptake. After 24 h treatment in cells, 100 nM Angptl3 stimulated a 2-fold induction of fructose uptake (Figure 2I). Importantly, other recombinant proteins generated using the same method, including Tsukushin (Tsk) and the closely related family member Angptl4, did not induce fructose uptake at 100 nM (Figure 2I). Additionally, 100 nM insulin did not induce fructose uptake under the same conditions (Figure 2J). Lastly, 100 nM Angptl3 did not induce glucose uptake in AML12 cells under the same conditions (Figure S1J). Thus, these results show that ANGPTL3 is a regulator of fructose uptake in hepatocytes, likely through a cell-autonomous feedforward mechanism.

### Circulating ANGPTL3 levels are elevated in response to dietary fructose

Prior studies have shown that human ANGPTL3 serum levels correlate with MASLD and MASH.^52^ Studies in rhesus macaques have shown that dietary fructose increases circulating levels of ANGPTL3 by 30%–40%.^53^ To investigate the role of fructose-induced regulation of fructose uptake without changing the overall diet composition, we fed mice a standard chow diet and supplemented the drinking water with 30% fructose or glucose.^54^ When analyzing circulating ANGPTL3 levels, we observed an elevation in the fructose water group compared to glucose controls (Figure 2K). Mice consuming fructose gained less weight compared to mice consuming glucose (Figure 2L). Water intake was higher when fed with glucose compared with fructose likely due to the innate preference of taste (Figure 2M). Weekly monitoring of blood glucose levels revealed no significant difference between mice given fructose- or glucose-supplemented water (Figure 2N). These findings suggest that fructose leads to elevated ANGPTL3 secretion, independent of overall caloric intake, glucose levels, or body weight.

### ANGPTL3 knockdown reduces cellular fructose metabolism

**Figure 3.**
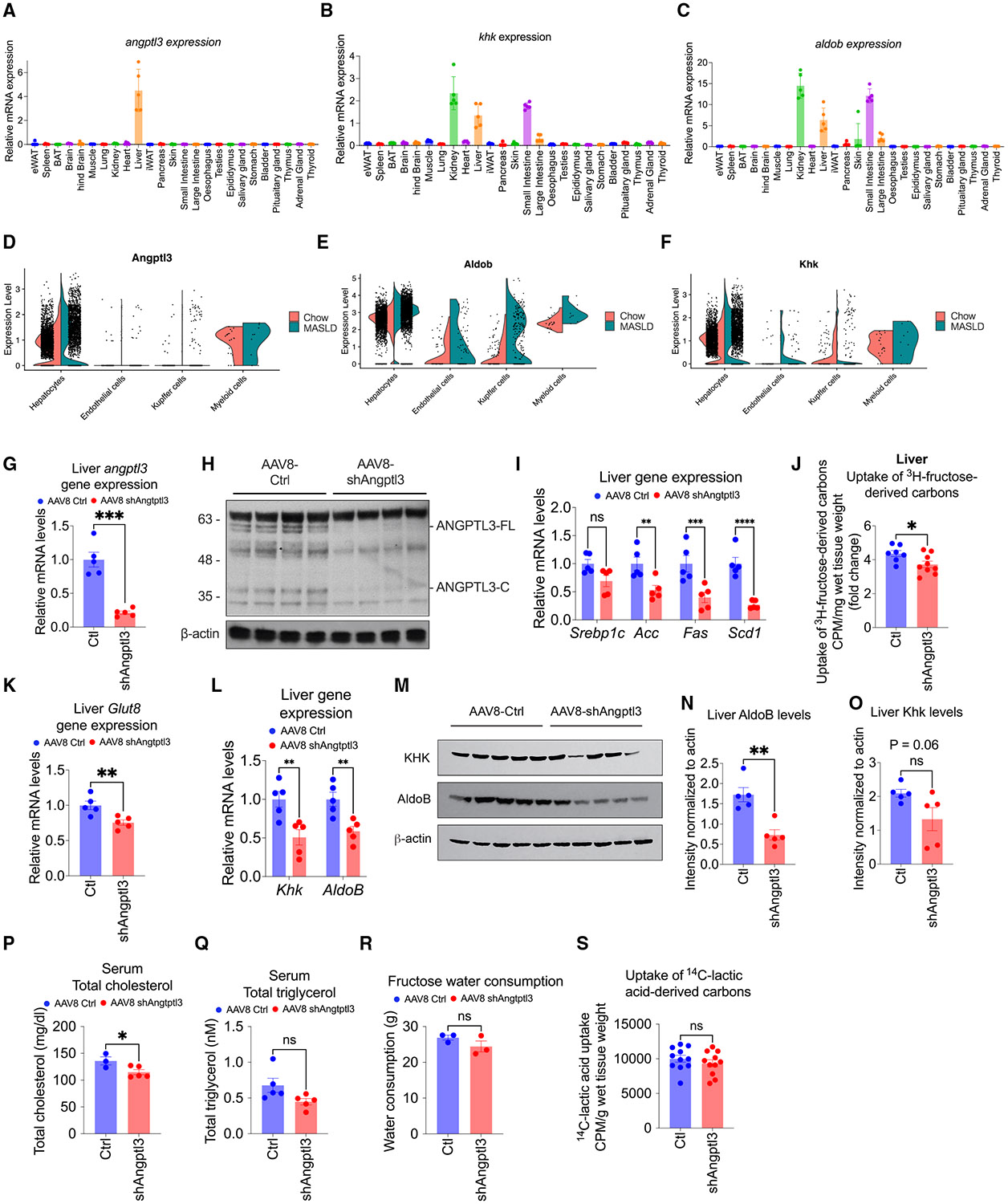
ANGPTL3 loss of function leads to reduced fructolysis enzyme levels (A) Relative gene expression analysis of angptl3 in male mouse organs (*N* = 5 mice per group). (B) Relative gene expression analysis of *khk (khk-a and khk-c)* in male mouse organs (*N* = 5 mice per group). (C) Relative gene expression analysis of *aldob* in male mouse organs (*N* = 5 mice per group). (D–F) Gene expression levels of *angptl3, aldob,* and *khk (khk-a and khk-c)* in male chow and MASLD mouse livers measured by single-cell RNA sequencing (*N* = 5 mice per group). (G) Relative gene expression analysis of *angptl3* in livers from AAV8-Ctl- and AAV8-shAngptl3-treated male mice (*N* = 5 mice per group, repeated in two independent cohorts). (H) Representative western blot of the liver from AAV8-Ctl- and AAV8-shAngptl3-treated male mice using an anti-Angptl3 antibody (*N* = 4 mice per group). (I) Relative gene expression analysis of hepatic lipogenesis genes from AAV8-Ctl- and AAV8-shAngptl3-treated male mice (*N* = 5 mice per group). (J) Uptake of [3H]-fructose-derived carbons in the liver of male mice fed with MASLD diet for 1 week (*N* = 5 mice per group). (K) Relative gene expression levels of Glut8 in livers from AAV8-Ctl- and AAV8-shAngptl3-treated male mice (*N* = 5 mice per group). (L) Relative gene expression levels of fructolysis enzyme genes in livers from AAV8-Ctl- and AAV8-shAngptl3-treated male mice (*N* = 5 mice per group). (M) Representative western blot of fructolysis enzymes in livers from AAV8-Ctl- and AAV8-shAngptl3-treated male mice (*N* = 5 mice per group). (N) Quantification of aldob protein levels relative to β-actin in (J) (*N* = 5 mice per group). (O) Quantification of Khk protein levels relative to β-actin in (J) (*N* = 5 mice per group). (P) Total cholesterol levels in the serum from AAV8-Ctl- and AAV8-shAngptl3-treated male mice (*N* = 3–5 mice per group). (Q) Total triglycerol levels in the serum from AAV8-Ctl- and AAV8-shAngptl3-treated male mice (*N* = 5 mice per group). (R) Water consumption in AAV8-Ctl- and AAV8-shAngptl3-treated male mice fed fructose in drinking water (*N* = 3 mice per group). (S) *In vivo* lactic acid uptake in the liver measured by [14C]-lactate incorporation from male mice fed with MASLD diet for 1 week (*N* = 11 mice per group). Data are presented as mean ± SEM. *p < 0.05, **p < 0.01, ***p < 0.001 by two-tailed Student’s t test (G, J, K, N, O, P, Q, R, and S) or two-way ANOVA (I and L).

Human ANGPTL3 is predominantly expressed in hepatocytes^55^ in the liver (Figure S3A). In mice, *angptl3* expression is restricted to the liver (Figure 3A), while the fructolysis enzymes *khk* (Figure 3B) and *aldob* (Figure 3C) are also expressed in the kidney and small intestine. ANGPTL3’s role in liver fructose metabolism under high-fat, high-fructose diets remains unexplored despite its known effects on lipid metabolism and atherogenic lipoproteins.^42^ By analyzing single-cell transcriptomic data, we confirm that angptl3 is co-expressed with the fructolysis enzymes *khk* and *aldob* as well as transporters *glut2* and *glut8* in several hepatocyte subpopulations (Figures S3B-S3G). We first tested whether *glut2* or *glut8* expression in hepatocytes was elevated in MASLD, but we did not observe any significant increase in expression (Figures S3H and S3I). Interestingly, in MASLD hepatocytes compared to chow-fed controls, *angptl3* expression was upregulated (Figures 2G and 3D) alongside the key fructose metabolism gene *aldob* (Figure 3E), while *khk* (Figure 3F) expression was not significant. This suggests a possible concurrent upregulation of fructose metabolism and ANGPTL3 in MASLD.

To study the effect of circulating ANGPTL3 on fructose metabolism *in vivo*, we transduced mice with viral expression vectors to lower circulating ANGPTL3 levels, followed by MASLD diet feeding. Five-week-old mice were i.v. injected with 2 × 10^11^ virus particles of adeno-associated virus serotype 8 (AAV8) expressing either shAngptl3 or scrambled shAngptl3 (Ctrl), both with a GFP tag. As predicted, the AAV8-shAngptl3 resulted in knockdown of hepatic *angptl3* (Figure 3G) without significantly changing the other angiopoietin-like family members *angptl4* and *angptl8* (Figure S3J). ANGPTL3 protein levels were also reduced in circulation, as determined by the detection of full-length and C-terminal ANGPTL3 in plasma using an ANGPTL3 antibody^56^ (Figure 3H). The shAngptl3 mice had a blunted lipogenesis gene expression, including *srebp1c*, *acc*, *fas*, and *scd1*, which was significantly different after 1 week of MASLD diet feeding (Figure 3I). Notably, Angptl3 knockdown resulted in a significant reduction in hepatic uptake of fructose-derived carbons *in vivo* (Figure 3J). To determine whether Glut8 was regulated by Angptl3, we measured *Glut8* gene expression levels and found that *Glut8* was downregulated in mice lacking Angptl3 (Figure 3K). In contrast, *Glut2*, *Glut5*, and *Glut9* expression levels were not affected (Figure S3K). These data suggest that GLUT8 is a hepatic fructose transporter that is under regulation by ANGPTL3. Under reduced fructose uptake conditions, the downstream fructolysis enzymes are expected to be downregulated, reflecting a reduced demand for their activity. Indeed, Angptl3 knockdown also resulted in lower levels of the fructolysis enzymes *khk* and aldolase B (*aldob*) at both the transcript (Figure 3L) and protein levels (Figures 3M-3O). In addition to altered fructose uptake, ANGPTL3 knockdown mice also exhibited lower circulating lipid levels, including a significantly reduced total cholesterol level (Figure 3P) and a trend toward lower serum triglyceride levels (Figure 3Q). Fructose consumption, assessed by measuring water intake over 1 week on fructose-containing water, did not differ between control and ANGPTL3 knockdown mice at this time point (Figure 3R). To evaluate the uptake of one of the fructose-derived metabolites, we administered ^14^C-lactic acid *in vivo* and found no significant difference in hepatic uptake of lactate or lactate-derived carbons between control and ANGPTL3 knockdown mice (Figure 3S), measured at the same time point when differences in ^3^H-fructose incorporation were observed. Together, these findings suggest that, while ANGPTL3 modulates hepatic uptake of fructose-derived carbons, it does not influence fructose ingestion or hepatic uptake of lactate.

### Cellular fructose uptake is independent of ANGPTL3’s LPL inhibitory activity

**Figure 4.**
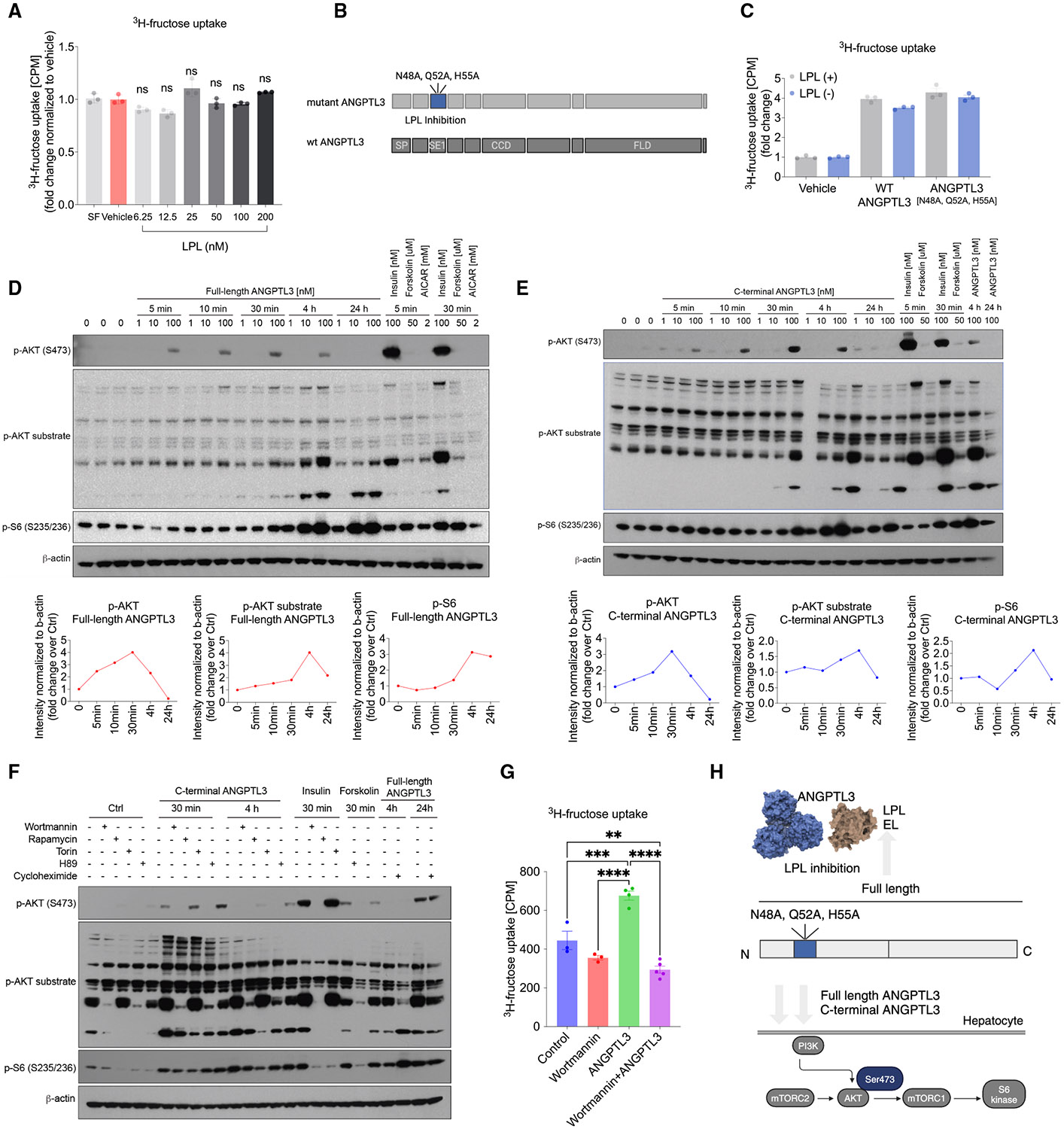
ANGPTL3 activates the AKT pathway independently of its interaction with LPL (A) [3H]-fructose uptake in AML12 cells after treatment with increasing concentrations of LPL in the presence of 100 nM human ANGPTL3 (*N* = 3 samples per group). (B) Schematic of generated point mutants of ANGPTL3. (C) [3H]-fructose uptake in AML12 cells after treatment with 100 nM wild-type or catalytically dead mutant of human ANGPTL3 in the presence or absence of LPL (*N* = 3 samples per group). (D) Representative western blot and quantification of *p*-AKT, *p*-AKT substrate, p-S6 in HepG2 cells treated with indicated doses of full-length human ANGPTL3, and positive controls showing the intracellular signaling pathways over time (*N* = 1 sample per group, repeated in two independent experiments). (E) Representative western blot and quantification of *p*-AKT, *p*-AKT substrate, p-S6 in HepG2 cells treated with indicated doses of C-terminal human ANGPTL3, and positive controls showing the intracellular signaling pathways over time (*N* = 1 sample per group, repeated in two independent experiments). (F) Representative western blot of *p*-AKT; *p*-AKT substrate; p-S6 induced by C-terminal human ANGPTL3 (100 nM); or positive controls in HepG2 cells pretreated with PI3K inhibitors wortmannin (1 μM), mTORC1 inhibitor rapamycin (100 nM), mTORC1/2 dual inhibitor torin (100 nM), PKA inhibitor H89 (10 μM), or the translation inhibitor cycloheximide (10 μM) (*N* = 1 sample per group). (G) [3H]-fructose uptake in AML12 cells after treatment with wortmannin with or without ANGPTL3 (*N* = 8–9 samples per group). (H) Schematic of ANGPTL3-induced cellular signaling pathways in hepatocytes. Data are presented as mean ± SEM. *p < 0.05, **p < 0.01, ***p < 0.001, ****p < 0.0001 by one-way ANOVA (A, C, and G).

ANGPTL3 was first identified in 1999^38^ and has since then been extensively studied as an inhibitor of lipoprotein lipase (LPL) and endothelial lipase to control circulating lipids.^52,57-59^ To investigate whether the regulation of fructose uptake was dependent on the interaction between ANGPTL3 and LPL, we exogenously added LPL in the presence of ANGPTL3 to hepatocytes. We observed that the presence of LPL did not alter the ability of ANGPTL3 to facilitate fructose uptake, as consistent fructose uptake rates in the presence of recombinant ANGPTL3 were observed, irrespective of LPL titration (Figure 4A). To further corroborate these results, we introduced a targeted triplemutant in the N-terminal part of ANGPTL3 (N48A, Q52A, and H55A) targeting three polar residues required for LPL inhibition (Figure 4B).^60^ The mutated ANGPTL3, deficient in LPL inhibitory function, showed no decrease in fructose uptake capability when compared to the wild-type protein (Figure 4C). This mutation-induced disruption of the ANGPTL3-LPL interaction is consistent with an LPL-independent pathway of ANGPTL3 in the regulating fructose uptake in hepatocytes. Overall, these findings suggest that ANGPTL3-mediated stimulation of fructose uptake occurs through a mechanism distinct from its LPL inhibitory activity.

### ANGPTL3 activates the AKT signaling pathway in hepatocytes

Since ANGPTL3 regulates cellular fructose uptake through an LPL-independent pathway, we investigated whether ANGPTL3 activates any signaling pathways in cells. Using full-length ANGPTL3, we observed that it induced p-AKT signaling at S473 in a time- and dose-dependent manner, with activation detectable as early as 5 min and peaking at 30 min post-treatment (Figure 4D). The downstream substrates of p-AKT, such as p-S6 at S235/236, were also activated, though their induction began at 30 min, later than p-AKT activation. In contrast, p-PKA substrates were not induced until 4 h post-treatment, suggesting that this is a secondary effect following the activation of the p-AKT pathway (Figure S4A). ANGPTL3 is reported to be cleaved into two fragments *in vivo* to a biologically active N-terminal fragment and an inactive C-terminal fragment.^61^ Given that the fructose-stimulatory effect of ANGPTL3 was independent of the N-terminally located LPL binding site, we next tested whether the C-terminal fragment was sufficient to induce p-AKT signaling. Indeed, the C-terminal ANGPTL3 fragment induced p-AKT signaling also at 100 nM after 5 min of treatment and caused sustained signaling up to 4 h (Figure 4E). At later time points, C-terminal ANGPTL3 also induced phosphorylation of AKT substrates, S6, and PKA substrates (Figures 4E and B). Importantly, inhibiting PI3K signaling using wortmannin and mTORC2-dependent signaling with Torin completely blunted the effect of ANGPTL3 on AKT signaling (Figures 4F, S4D, and S4E), while p-PKA signaling was inhibited by H89 inhibitor at 4 h but not 30 min (Figure S4C), suggesting that the PI3K-AKT pathway is a proximal pathway induced by ANGPTL3. These data highlight a novel mechanism of ANGPTL3 in regulating the p-AKT signaling pathway. We also found that wortmannin significantly reduced ANGPTL3-stimulated cellular fructose uptake and showed a trend toward lowering basal fructose uptake. These results are consistent with the involvement of the PI3K pathway in ANGPTL3-mediated fructose regulation (Figure 4G). While we observed a trend toward increased *Glut8* expression following ANGPTL3 treatment, LPL inhibition did not alter *Glut8* expression in hepatocytes (Figure S4F). In contrast, LPL inhibition using Gsk264220A or ANGPTL3 recombinant protein treatment increased *Srebp1c* expression as expected (Figure S4G). In conclusion, our study identifies ANGPTL3 as a novel regulator of hepatic fructose uptake, independent of its known function as an LPL inhibitor. ANGPTL3 also activates the PI3K/AKT pathway via both its full-length protein and the C-terminal fragment, providing important insights into the understanding of fructose regulation (Figure 4H).

## DISCUSSION

Fructose is a natural hexose that is metabolized and used as an energy substrate. However, high fructose ingestion is correlated with induced hepatic lipid accumulation and transcriptional activation of lipid synthesis genes.^44,62,63^ While the regulation of glucose metabolism has been extensively studied, how fructose transport and metabolism are regulated is far less studied. Given that MASLD is now estimated to affect 25% of the global population, identifying mechanisms by which fructose metabolism can be controlled is of high priority.^64^

Here, we find that uptake of fructose and fructose-derived carbons is upregulated in MASLD and that ANGPTL3 increases fructose uptake into hepatocytes. Interestingly, prior to our work, there was evidence in the literature of fructose transport in the intestine being a regulated process. Exposure to luminal fructose for 14 days in neonatal rats increased GLUT5 expression and intestinal fructose absorption, analogous to the increased fructose transport seen in hepatocytes in our study.^65^ Similarly, the intracellular protein TXNIP has been shown to not only regulate glucose transport but also induce intestinal fructose uptake in the diabetic state.^66^ Studies on the regulation of the expression of hexose transporters in MASLD and NASH have been limited. In one human study, NASH was shown to increase the expression of *Glut*1, -3, -5, -6, -7, -8, -9, -10, -11, -12, and -13, but no change in expression was seen in livers diagnosed with simple steatosis.^67^ Our mouse model resembles steatosis without fibrosis^43^ and is thus consistent with no changes in *Glut* transcript levels. Interestingly, insulin has been reported to result in translocation of GLUT8 from intracellular compartments to the cell surface in murine blastocysts where GLUT8 regulates glucose uptake.^68,69^ In contrast, we do not observe any increase in hepatic fructose uptake by insulin, arguing for a specific mechanism to regulate fructose uptake. Interestingly, a recent study demonstrated reduced hepatic triglycerides, inflammation, and fibrosis upon hepatocyte-specific silencing of GLUT8, but fructose uptake was not measured under these conditions.^49^ Our loss-of-function studies show reduced uptake of hepatic fructose-derived carbons in liver-specific GLUT8-KO mice, suggesting that endogenous GLUT8 contributes to the metabolism of fructose into the liver. The 50% reduction in *in vivo* fructose uptake observed in liver-specific GLUT8-KO mice is consistent with that GLUT8-mediated transport is not the sole pathway for hepatic fructose uptake. However, despite GLUT8’s low liver expression, prior studies^33,49^ and our study demonstrate that there is still a significant functional effect in GLUT8-deficient mice, even without elevated GLUT8 levels. The metabolic conditions in MASLD may favor a focus on downstream pathways of fructose metabolism rather than directly increasing transporter expression. There is also a need to better understand the relationship between GLUT8 and MASLD. Upon ANGPTL3 knockdown, we observe lower GLUT8 expression, possibly reflecting the reduced need for fructose uptake. While both MASLD and ANPTPL3 knockdown models can offer insights into ANGPTL3’s role, they may not necessarily show exact opposite phenotypes due to the complex nature of MASLD. The broader metabolic and environmental changes in MASLD could lead to distinct or overlapping but not fully opposite phenotypic outcomes. Future studies should explore these mechanisms to better understand the relationship between ANGPTL3 and GLUT8 in MASLD. The role, if any, of ANGPTL3 on intestinal fructose transport remains to be determined.

ANGPTL3 is known to be cleaved into a biologically active N-terminal fragment and a C-terminal fragment. Previous studies have primarily focused on the N-terminal fragment given its role in inhibiting lipoprotein and endothelial lipases leading to elevated plasma triglycerides. In addition, the C-terminal fragment has been reported to mediate angiogenesis.^70,71^ Our findings reveal a novel function for ANGPTL3 in activating the PI3K-AKT signaling pathway, a previously unrecognized bioactivity. We demonstrated that ANGPTL3 induces AKT activation, leading to increased phosphorylation of AKT and its substrates. This signaling cascade appears to be critical, as inhibition of PI3K and mTORC2 pathways completely blocked ANGPTL3’s effects on AKT signaling, and inhibiting PI3K signaling reduces fructose uptake. Despite extensive knowledge of ANGPTL3’s role in lipid metabolism, the conditions under which it is released and the specific receptors mediating its effects remain areas requiring further investigation to fully understand its regulatory mechanisms.^72^ This new mechanism for control of fructose transport might be useful to develop targeted therapies against fructose-induced hepatic steatosis.

### Limitations of the study

There are limitations of this work. One limitation is that the measured ^3^H-fructose uptake reflects total ^3^H-carbons, including both fructose and its metabolites. Our results confirm findings consistent with other studies that employed ^13^C-fructose.^16^ We selected time points within 7 min, allowing ^3^H uptake to serve as an indicator of fructose uptake. However, future studies should consider using stable isotopes, such as ^13^C, to differentiate labeled fructose from its metabolites. Furthermore, although we demonstrate that ANGPTL3’s involvement in fructose uptake is independent of LPL inhibition, we did not test this *in vivo*, highlighting a valuable direction for future research. The study of ANGPTL3 was done in the presence of fructose in the drinking water. In our comparison of healthy mice with fatty liver using MASLD and chow diets, the use of a chow control diet was a limitation. Only male mice were used in this study. The role of ANGPTL3 in female mice remains to be determined. Additionally, it is possible that there is a cell surface receptor that mediates the effects of ANGPTL3-C on fructose uptake in hepatocytes.

## STAR★METHODS

### EXPERIMENTAL MODEL AND STUDY PARTICIPANT DETAILS

#### Animal studies

Animal experiments were performed per procedures approved by the Institutional Animal Care and Use Committee of the Stanford Animal Care and Use Committee (APLAC) protocol number #32982. Wildtype C57BL/6J, B6.129X1-Slc2a8^tm1Thor^/J, and B6.Cg-Speer6-ps1Tg(Alb-cre)21Mgn/J mice were purchased from Jackson Laboratory. The MASLD diet (ResearchDiet, Cat# D09100310) contains 40% kcal from fat (palm oil), 20% kcal from fructose, and 2% cholesterol, and has been shown to induce fatty liver in rodent models.^75^ Unless otherwise stated, mice were housed in a temperature-controlled (20°C–22°C) room on a 12-h light/dark cycle (7 a.m.–7 p.m.). All experiments were performed with age-matched male mice housed in groups of five unless stated otherwise.

#### Culture of hepatocyte cell lines

AML12 mouse hepatocytes (#CRL-2254) were purchased from ATCC and cultured in DMEM/F12 medium (Gibco) supplemented with 10% FBS, 15 mM HEPES, 5.5 mg/mL transferrin, 40 ng/mL dexamethasone, 5 ng/mL selenium, and 10 mg/mL insulin. HepG2 (#HB-8065) and Expi293F cells (ThermoFisher #Cat#A14527) were cultured following the manufacturer’s guidelines. All cells were cultured in a humidified atmosphere with 8% CO2 for Expi293F cells and 5% CO2 for others, at 37°C.

#### Isolation and culture of primary mouse hepatocytes

Primary hepatocyte isolation was performed as previously described (Jung et al., 2020). Briefly, mice were euthanized, and livers were perfused in HBSS buffer supplemented with 0.4 g/L KCl, 1 g/L glucose, 2.1 g/L sodium bicarbonate, and 0.2 g/L EDTA (perfusion buffer) for 5–10 min. Collagenase digestion was then conducted for 10–15 min at 37 C. After being dissociated from the digested livers, cells were suspended in Williams Medium E supplemented with 10% FBS, 2 mM sodium pyruvate, 1 mM dexamethasone, and 100 nM insulin (plating medium) on ice. The cell suspension was filtered through 70 mm strainer and centrifuged at 50g for 5 min. The resulting pellet was resuspended in 40 mL of plating media and divided into four tubes, each containing 10 mL. Two tubes were mixed with 10 mL of 25% Percoll, and the other two with 10 mL of 90% Percoll. One tube from each set was centrifuged at 100g, and the other at 1,000g. After washing, hepatocytes were washed, resuspended, and seeded on collagen-coated plates. 4 h after seeding, hepatocytes were washed with PBS, and cultured in Williams E supplemented with 0.2% BSA, 2 mM sodium pyruvate, 0.1mM dexamethasone (maintenance medium).

### METHOD DETAILS

#### *In vivo* fructose experiments

Mice were fasted for 1h and injected i.p. with ^3^H-fructose at 100 μCi/kg (2g/kg) body weight based on prior studies^45^ to result in fructose and fructose-derived carbons in the circulation of 0.2 mM. 1 μCi/μL 3H-fructose stock was diluted in saline to generate a 120 μL injection volume per mouse. After 30 min, mice were euthanized. Blood was collected by cardiac puncture and subsequently centrifuged to collect serum. Wet tissue weights were recorded and then homogenized in 1% SDS for liquid scintillation counting. Data are expressed as CPM/mg wet weight for organs and CPM/μL for serum.

#### *In vivo* lactic acid experiments

Mice were fasted for 1h and injected i.p. with ^14^C-lactic acid sodium at 2.5 μCi/mouse. 0.1 mCi/mL ^14^C-lactic acid sodium stock was diluted in saline to generate a 100 μL injection volume per mouse. After 5 min, the mice were euthanized. Wet tissue weights were recorded and then homogenized in 1% SDS for liquid scintillation counting. Data are expressed as CPM/mg wet weight for organs.

#### *In vitro* fructose experiments

Cells were washed with KRH buffer and starved for 3 h in 0.5% BSA in KRH buffer. Treatments at specific concentrations were added at designated times. Cells were treated with a mix of 0.005 mM fructose and 4 μCi/mL 3H-fructose for 10 min followed by three washes with ice-cold KRH buffer. The cells were then lysed in 0.1% SDS, and radioactivity was quantified using liquid scintillation counting.

#### *In vitro* LPL competition

Recombinant versions of wild-type and LPL-mutant ANGPTL3 were expressed and purified as described below. Recombinant LPL was purchased from R&D Systems (#9888-LL-100) and dialyzed to KRH buffer to remove CHAPS detergent from the protein storage buffer. AML12 cells were washed with KRH buffer and starved for 16 h in starvation media (DMEM/F12 medium (Gibco) supplemented with 15 mM HEPES, 5.5 mg/mL transferrin, 40 ng/mL dexamethasone, 5 ng/mL selenium) with 100 nM recombinant ANGPTL3 and dialyzed LPL. Then, *in vitro* fructose uptake was performed as described above.

#### Expression and purification of recombinant proteins

The recombinant proteins used in this study were generated by transient transfection of DNA plasmids into Expi293F cells. All plasmids (Angptl4, Tsukushin, wild-type Angptl3, ANGPTL3 LPL mutant, N-terminal ANGPTL3, and C-terminal ANGPTL3) were verified by sequencing. Recombinant proteins (Angptl4, Tsukushin, wild-type Angptl3, ANGPTL3 LPL mutant, N-terminal ANGPTL3, and C-terminal ANGPTL3) with C-terminal flag or his tags were produced and purified from mammalian Expi293 cells using large scale transient DNA transfection. Proteins were purified using magnetic beads or columns according to the manufacturer’s descriptions and buffer exchanged to PBS. Protein purity and integrity was assessed with SDS page, Superdex200 size exclusion column and endotoxin assay. All proteins were aliquoted and stored in −80°C and not used for more than three freeze-thaws.

#### Gene expression analysis

Total RNA from cultured cells or tissues was isolated using TRIzol (Thermo Fischer Scientific) and Rneasy mini kits (QIAGEN). RNA was reverse transcribed using the ABI high-capacity cDNA synthesis kit. For q-RT-pcr analysis, cDNA, primers and SYBR-green fluorescent dye (ABI) was used. Relative mRNA expression was determined by normalization with Cyclophilin levels using the ΔΔCt method. Primer sequences used are described in the key resources table.

#### Western blots and molecular analyses

For western blotting, homogenized tissues or whole cell lysates, samples were lysed in RIPA buffer containing protease inhibitor cocktail (Roche) and phosphatase inhibitor cocktail (Roche), prepared in 4X LDS Sample Buffer (Invitrogen) and separated by SDS-PAGE and transferred to Immobilon 0.45μm membranes (Millipore). Protein kinase array (R&D systems ARY003B) was performed according to the manufacturers’ description. For Western blotting of plasma samples, 1 μL of plasma was prepared containing 2X sample buffer (Invitrogen) with reducing agent, boiled, and analyzed using Western blot against indicated antibodies.

#### Lipogenesis assay

Primary hepatocytes or AML12 were washed with warm PBS twice and starved in serum-free DMEM overnight. Indicated concentrations of insulin with or without Ism1 were added at the same time. The following day, a mixture of 10 μM cold acetate and 2 μCi 3H-Acetate was added to each well and cells were incubated for another 4 h. Cells were washed with PBS twice and lysed using 0.1 N hydrogen chloride. Lipids were extracted by 2:1 chloroform-methanol (v/v). After 10 min centrifugation at 3000 x g, lower phase was transferred to scintillation vial and 3H activity was measured by liquid scintillation counting.

#### Proteomics of the hepatocyte secretome

Samples were run on a 4–12% SDS-PAGE gel and resolved by Coomassie staining. Gel bands were excised out in a 1.5 mL Eppendorf tubes and then cut in 1 × 1 mm squares. The excised gel pieces were then reduced with 5 mM DTT, 50 mM ammonium bicarbonate at 55°C for 30 min. Residual solvent was removed and alkylation was performed using 10 mM acrylamide in 50 mM ammonium bicarbonate for 30 min at room temperature. The gel pieces were rinsed 2 times with 50% acetonitrile, 50 mM ammonium bicarbonate and placed in a speed vac for 5 min. Digestion was performed with Trypsin/LysC (Promega) in the presence of 0.02% protease max (Promega) in both a standard overnight digest at 37°C. Samples were centrifuged and the solvent including peptides was collected and further peptide extraction was performed by the addition of 60% acetonitrile, 39.9% water, 0.1% formic acid and incubation for 10–15 min. The peptide pools were dried in a speed vac. Samples were reconstituted in 12μL reconstitution buffer (2% acetonitrile with 0.1% Formic acid) and 3μL (100ng) of it was injected on the instrument.

Mass spectrometry experiments were performed using a Q Exactive HF-X Hybrid Quadrupole - Orbitrap mass spectrometer (Thermo Scientific, San Jose, CA) with liquid chromatography using a Nanoacquity UPLC (Waters Corporation, Milford, MA). For a typical LCMS experiment, a flow rate of 600 nL/min was used, where mobile phase A was 0.2% formic acid in water and mobile phase B was 0.2% formic acid in acetonitrile. Analytical columns were prepared in-house with an I.D. of 100 μm packed with Magic 1.8 μm 120Å UChrom C18 stationary phase (nanoLCMS Solutions) to a length of ~25 cm. Peptides were directly injected onto the analytical column using a gradient (2–45% B, followed by a high-B wash) of 80min. The mass spectrometer was operated in a data dependent fashion using HCD fragmentation for MS/MS spectra generation. For data analysis, the.RAW data files were processed using Byonic v3.2.0 (Protein Metrics, San Carlos, CA) to identify peptides and infer proteins using Mus musculus database from Uniprot. Proteolysis with was assumed to be semi-specific allowing for N-ragged cleavage with up to two missed cleavage sites. Precursor and fragment mass accuracies were held within 12 ppm. Proteins were held to a false discovery rate of 1%, using standard approaches. Mass spectrometry data is supplied in Table S1. The raw mass spectrometry data has been deposited at ProteomeXchange Consortium under accession number. PXD054506 (JPST003248).

### QUANTIFICATION AND STATISTICAL ANALYSIS

Student’s t test was used for single comparisons. Significant differences between two groups (*p < 0.05, **p < 0.01, ***p < 0.001) were evaluated using a two-tailed, unpaired t test when the sample groups displayed a normal distribution and comparable variance. One-way ANOVA was used for comparisons among three or more groups (*p < 0.05, **p < 0.01, ***p < 0.001). two-way ANOVA was used for group comparisons involving two or more factors, with repeated measures applied where appropriate (*p < 0.05, **p < 0.01, ***p < 0.001). The statistical details of experiments can be found in the figures and figure legends. Mice were randomly assigned to treatment groups for *in vivo* studies. All data are presented as mean ± S.E.M as described in the figure legend. Specific details for *n* values are noted in each figure legend.

## Supplementary Material

1

2

Supplemental information can be found online at https://doi.org/10.1016/j.celrep.2025.115962.

## Figures and Tables

**Table:** KEY RESOURCES TABLE

REAGENT or RESOURCE	SOURCE	IDENTIFIER
Antibodies		
Mouse Angiopoietin-like Protein 3/ANGPTL3 Antibody	R&D Systems	Cat# AF136; RRID: 354753
Anti-KHK antibody	Sigma-Aldrich	Cat# HPA007040; RRID: AB_1079185
ALDOB Polyclonal Antibody	Thermo Fisher	Cat# 18065-1-AP; RRID: AB_2273968
Mouse monoclonal anti-beta Actin [AC-15] (HRP)	Abcam	Cat# AB49900; RRID: AB_86749
Donkey anti-rabbit IgG (HRP)	Cytiva (GE)	CAT#NA934; RRID: AB_772206
Rabbit anti-goat IgG HRP-conjugated Antibody	R&D Systems	Cat# HAF017; RRID: AB_562588
Bacterial and virus strains		
One Shot TOP10 Chemically Competent E. coli	Thermo Fisher Scientific	Cat# C404010
AAV8-GFP-mANGPTL3-shRNA	This paper, UPenn Vector Core	N/A
AAV8-GFP-scrmb-shRNA	This paper, UPenn Vector Core	N/A
Chemicals, peptides, and recombinant proteins		
Mouse recombinant ANGPTL3-flag protein	This paper	N/A
Mouse recombinant ANGPTL3-his protein	This paper	N/A
Mouse recombinant ANGPTL4-flag protein	This paper	N/A
Mouse recombinant TSK-flag protein	This paper	N/A
Human recombinant insulin	Millipore Sigma	Cat# 91077C
Bovine Serum Albumin	Millipore Sigma	Cat# A7906; CAS:9048-46-8
Recombinant Human Lipoprotein Lipase/LPL Protein	R&D Systems	Cat# 9888-LL-100
2-[1,2-3H (N)]-Deoxy-D-glucose	Perkin-Elmer	Cat# NET328A001MC
D-[6-^3^H(N)]-Glucose	PerkinElmer	Cat# NET100C001MC
D-Fructose [3H(G)]	American Radiolabeled Chemicals	Cat# ART0329
Lactic acid, l-[14c(u)] sodium salt	American Radiolabeled Chemicals	Cat# ARC0593-50 μCi
Ketohexokinase inhibitor 1	Selleckchem	Cat# S0808
Wortmannin	Selleckchem	Cat# S2758
Cytochalasin B	Sigma-Aldrich	Cat# C6762
2x SYBR Green qPCR master mix	Bimake	Cat# B21203
Trizol	Thermo Fisher	Cat# 15-596-026
High-capacity cDNA Reverse Transcription kit	Biosystems	Cat# 4368814
Expi293 Expression medium	Thermo Fisher	Cat# A1435101
Opti-MEM reduced serum media	Thermo Fisher	Cat# 31985062
DMEM/F12 (1:1)	Thermo Fisher	Cat# 11320082
DMEM/F12 + Glutamax	Thermo Fisher	Cat# 10565-042
MEM	Thermo Fisher	Cat# 11095080
HBSS buffer	Gibco	Cat# 14175-095
HBSS buffer	Gibco	Cat# 14175-095
Potassium chloride	Sigma	Cat# P5405
Sodium bicarbonate	Sigma	Cat# S6297
UltraPure 0.5 M EDTA, pH 8.0	Invitrogen	Cat# 15575-038
Williams E media	Quality Biological	Cat# 112-033-101
Corning regular fetal bovine serum	Corning	Cat# 35-010-CV
DMEM high glucose	Sigma	Cat# D6429
HEPES buffered saline	Sigma-Aldrich	Cat# 51558
Sodium pyruvate	Thermo Fisher	Cat# 11360070
Dexamethasone	Sigma	Cat# D1756
Human transferrin	Sigma	Cat# T-2252
Selenium	Fisher Scientific	Cat# US-ICP-034
Penicillin/streptomycin	Gibco	Cat# 15140-122
Collagenase IV	Sigma	Cat# C5138
PBS	Gibco	Cat#10010-023
10x PBS	Gibco	Cat#70011044
Percoll	Sigma	Cat# P1644
BSA	Sigma	Cat# A7906
Glutamax	Thermo Fisher	Cat# 35050061
Isopropanol	Fisher Chemical	Cat# BP2632-4
Rat tail Collagen I	Corning	Cat# 354236
Trypan blue stain (0.4%)	Invitrogen	Cat# T10282
D-(+)-Glucose	Sigma	Cat# 50-99-7
2-Deoxy-D-glucose	Sigma	Cat# D8375
Fructose	Fisher Scientific	Cat# AAA1771830
Trypsin/EDTA 0.25%	Gibco	Cat# 25200-056
Immobilon Crescendo Western HRP substrate	Millipore Sigma	Cat ## WBLUR0500
SuperSignal West Femto HP substrate	Thermo Scientific	Cat# 34095
SeeBlue Plus2 prestained standard	Invitrogen	Cat# LC5925
RIPA buffer (10x)	Cell Signaling	Cat# 9806S
NuPAGE LDS sample buffer (4x)	Invitrogen	Cat# NP0007
PhosSTOP	Roche	Cat# 04906837001
cOmplete Tablets	Roche	Cat# 04693124001
HEPES, pH7.4	Sigma-Aldrich	Cat# 51558
CaCl2	Sigma	Cat#100-43-52-4
KCl	Quality Biological	Cat# 112-033-101
GSK264220A	Cayman	Cat#13009
Critical commercial assays		
Mouse Angiopoietin-like 3 Immunoassay	R&D Systems	Cat# MANL30
Pierce BCA Protein Assay Kit	Thermo Fisher	Cat# 23225
Cholesterol Quantification Assay kit	Sigma-Aldrich	Cat# CS0005-1KT
Triglyceride Assay Kit	Abcam	Cat# ab65336
Deposited data		
Raw proteomics data	ProteomeXchange Consortium	PXD054506 (JPST003248)
Single cell data	(Coassolo et al.^44^)	Coassolo et al.^44^
Experimental models: cell lines		
AML12 cells	ATCC	Cat# CRL-2254; RRID: CVCL_0140
HepG2 cells	ATCC	Cat# HB-8065; RRID: CVCL_0027
Primary mouse hepatocytes	This paper	N/A
Expi293F Cells	Thermo Fisher Scientific	Cat# A14527
Experimental models: organisms/strains		
Mouse: C57BL/6J	Jackson Laboratory	Cat# 000664; RRID: IMSR_JAX:000664
Mouse: B6.129X1-Slc2a8^tm1Thor^/J	Jackson Laboratory	Cat# 032491; RRID: IMSR_JAX:032491
Mouse: B6.Cg-Speer6-ps1Tg(Alb-cre)21Mgn/J	Jackson Laboratory	Cat# 003574; RRID: IMSR_JAX:003574
Mouse: Glut8 liver-specific knockout	This paper	N/A
Oligonucleotides		
pAAV-GFP-U6-mANGPTL3-shRNA	Vector Biolabs	Cat# shAAV-252619
pAAV-GFP-U6-scrmb-shRNA	Vector Biolabs	N/A
Genotyping primers: Slc2a8 flox: CTG GAA GGC AGA GCC AAA G; ACC GTA GGG TCT GAG CAT CT	Jackson Laboratory	Cat# 032491; RRID: IMSR_JAX:032491
Genotyping primers: Alb-cre Wild type Forward: TGC AAA CAT CAC ATG CAC AC Mutant Forward: GAA GCA GAA GCT TAG GAA GAT GG Common: TTG GCC CCT TAC CAT AAC TG	Jackson Laboratory	Cat# 003574; RRID: IMSR_JAX:003574
Primers for q-RT-PCR: Mouse Slc2a1 CTCTGTCGGCCTCTTTGTTAAT; CCAGTTTGGAGAAGCCCATAAG	Primerbank	N/A
Mouse Slc2a2 ACTTGGAAGGATCAAAGCAATGT; CAGTCCTGAAATTAGCCCACAA	Primerbank	N/A
Mouse Slc2a3 ATGGGGACAACGAAGGTGAC; GTCTCAGGTGCATTGATGACTC	Primerbank	N/A
Mouse Slc2a4 CTCATGGGCCTAGCCAATGC; CCCTGATGTTAGCCCTGAGTA	Primerbank	N/A
Mouse Slc2a5 TCTCTTCCAACGTGGTCCCTA; GAGACTCCGAAGGCCAAACAG	Primerbank	N/A
Mouse Slc2a6 GCATCCCGGTGTACGTGTC; CAAGCCATCGCCAAGGTAG	Primerbank	N/A
Mouse Slc2a7 CCCTTCGTGACTGGCTTTG; TGGGTAGGCGATTTCCGAGAT	This paper	N/A
Mouse Slc2a8 CCCTTCGTGACTGGCTTTG; TGGGTAGGCGATTTCCGAGAT	Primerbank	N/A
Mouse Slc2a8 (for Slc2a8-KO mouse validation) TGGGAGATCAGAAAGGGACT; TGAAACTCAACAAGATATGGTG	This paper	N/A
Mouse Slc2a9 GCCCACGCTACCTTCTCTTTG; AACCAGATCGCATTGAGTCCA	Primerbank	N/A
Mouse Slc2a10 AGTTTCAGACAAGCAGGTTCC; GCATCTTCCAAGCAGACGGA	This paper	N/A
Mouse Slc2a12 AGGTCCCAGCATGTTTACGTT; GGGCTAATAGCGTTCTGATCTG	Primerbank	N/A
Mouse Slc5a9 CGGAGCTTGTGGCAATGGA; TGCACGGATGGATGACCAAAT	Primerbank	N/A
Mouse Slc5a10 TCGGAGCCTCACTCTTTGC; ATCCGCACGTACTTATCTGTCC	Primerbank	N/A
Mouse Slc45a2 AATGGAGATGCGGTCGTATCA; TATGGCACCCAAAATGTAGCC	Primerbank	N/A
Mouse Slc45a3 CGGCATTACCTATGTGCCAC; GCCCAGATAAAGGGTCTCCG	Primerbank	N/A
Mouse Slc45a4 CCCTCAACATACACGCCTTCT; CGTCTGGAACCAGTCACCT	Primerbank	N/A
Human Slc2a2 GGGCAATTATGATCTGTGGCA; TTCTGCTCACTCGATGCTTCT	Primerbank	N/A
Human Slc2a5 CGTGCCTGCGATCTTAATGG; GATACACCTGCACATATTCCCAC	Primerbank	N/A
Human Slc2a8 CCGGCATCTACAAGCCCTTC; ATAGAACATGACGGCGTTGAC	Primerbank	N/A
Human Slc2a9 GCCGTCTTCTCTGGTTTGGT; GCCCAACAGCAAAGTTGGAG	This paper	N/A
Mouse Angptl3 GAGGAGCAGCTAACCAACTTAAT; TCTGCATGTGCTGTTGACTTAAT	Primerbank	N/A
Mouse Angptl4 CATCCTGGGACGAGATGAACT; TGACAAGCGTTACCACAGGC	Primerbank	N/A
Mouse Angptl8 GACACTGTACGGAGACTAC; AGGTGGCTCTGCTTATCA	Tang et al. 2022^73^	N/A
Mouse Srebp1c GGAGCCATGGATTGCACATT; GGCCCGGGAAGTCACTGT	Jiang et al. 2021^74^	N/A
Mouse Acc CGCTCAGGTCACCAAAAAGAAT; GTCCCGGCCACATAACTGAT	Jiang et al. 2021^74^	N/A
Mouse Fas GGAGGTGGTGATAGCCGGTAT; TGGGTAATCCATAGAGCCCAG	Jiang et al. 2021^74^	N/A
Mouse Scd1 TTCTTGCGATACACTCTGGTGC; CGGGATTGAATGTTCTTGTCGT	Jiang et al. 2021^74^	N/A
Mouse Khk ATGTGGTGGACAAATACCCAGA; CAAGCAAGGAAAGGACAGTGC	Primerbank	N/A
Mouse Aldob TTGTGGTGGGCATCAAGTTG; AGCACGCCACTTCCCAAAG	Primerbank	N/A
Recombinant DNA		
Angptl3 expression plasmid	OriGene	MR207262
Angptl4 expression plasmid	OriGene	MR206437
Tsk expression plasmid	OriGene	MR218507
Software and algorithms		
GraphPad Prism version 10	GraphPad Software	RRID: SCR_002798
Adobe Illustrator	Adobe	RRID: SCR_010279
Other		
ExpiFectamine 293 Transfection Kit	Thermo Fisher	Cat# A14524
MASLD diet	ResearchDiet	Cat# D09100310

## Highlights

- Hepatocytes secrete ANGPTL3, which increases fructose-derived metabolite uptake
- Circulating levels of ANGPTL3 increase in response to fructose exposure
- ANGPTL3 knockdown lowers hepatic fructolysis enzyme levels
- ANGPTL3’s fructose regulation is independent of its LPL inhibitory function

## References

[1] Elliott SS, Keim NL, Stern JS, Teff K, and Havel PJ (2002). Fructose, weight gain, and the insulin resistance syndrome. Am. J. Clin. Nutr 76, 911–922. 10.1093/ajcn/76.5.911. 12399260

[2] Marriott BP, Cole N, and Lee E (2009). National estimates of dietary fructose intake increased from 1977 to 2004 in the United States. J. Nutr 139, 1228S–1235S. 10.3945/jn.108.098277. 19403716

[3] Tetri LH, Basaranoglu M, Brunt EM, Yerian LM, and Neuschwander-Tetri BA (2008). Severe NAFLD with hepatic necroinflammatory changes in mice fed trans fats and a high-fructose corn syrup equivalent. Am. J. Physiol. Gastrointest. Liver Physiol 295, G987–G995. 10.1152/ajpgi.90272.2008. 18772365 PMC4059366

[4] Im YR, Hunter H, de Gracia Hahn D, Duret A, Cheah Q, Dong J, Fairey M, Hjalmarsson C, Li A, Lim HK, (2021). A Systematic Review of Animal Models of NAFLD Finds High-Fat, High-Fructose Diets Most Closely Resemble Human NAFLD. Hepatol. Baltim. Md 74, 1884–1901. 10.1002/hep.31897. 33973269

[5] James J, Thomas P, Cavan D, and Kerr D (2004). Preventing childhood obesity by reducing consumption of carbonated drinks: cluster randomised controlled trial. BMJ 328, 1237. 10.1136/bmj.38077.458438.EE. 15107313 PMC416601

[6] Zivkovic AM, German JB, and Sanyal AJ (2007). Comparative review of diets for the metabolic syndrome: implications for nonalcoholic fatty liver disease. Am. J. Clin. Nutr 86, 285–300. 10.1093/ajcn/86.2.285. 17684197

[7] Zelber-Sagi S, Ratziu V, and Oren R (2011). Nutrition and physical activity in NAFLD: an overview of the epidemiological evidence. World J. Gastroenterol 17, 3377–3389. 10.3748/wjg.v17.i29.3377. 21876630 PMC3160564

[8] Yki-Järvinen H. (2010). Nutritional modulation of nonalcoholic fatty liver disease and insulin resistance: human data. Curr. Opin. Clin. Nutr. Metab. Care 13, 709–714. 10.1097/MCO.0b013e32833f4b34. 20842026

[9] Basaranoglu M, Basaranoglu G, Sabuncu T, and Sentürk H (2013). Fructose as a key player in the development of fatty liver disease. World J. Gastroenterol 19, 1166–1172. 10.3748/wjg.v19.i8.1166. 23482247 PMC3587472

[10] Thuy S, Ladurner R, Volynets V, Wagner S, Strahl S, Königsrainer A, Maier KP, Bischoff SC, and Bergheim I (2008). Nonalcoholic fatty liver disease in humans is associated with increased plasma endotoxin and plasminogen activator inhibitor 1 concentrations and with fructose intake. J. Nutr 138, 1452–1455. 10.1093/jn/138.8.1452. 18641190

[11] Hochuli M, Aeberli I, Weiss A, Hersberger M, Troxler H, Gerber PA, Spinas GA, and Berneis K (2014). Sugar-sweetened beverages with moderate amounts of fructose, but not sucrose, induce Fatty Acid synthesis in healthy young men: a randomized crossover study. J. Clin. Endocrinol. Metab 99, 2164–2172. 10.1210/jc.2013-3856. 24601726

[12] Baena M, Sangüesa G, Dávalos A, Latasa MJ, Sala-Vila A, Sánchez RM, Roglans N, Laguna JC, and Alegret M (2016). Fructose, but not glucose, impairs insulin signaling in the three major insulin-sensitive tissues. Sci. Rep 6, 1–15. 10.1038/srep26149. 27194405 PMC4872141

[13] Softic S, Gupta MK, Wang GX, Fujisaka S, O’Neill BT, Rao TN, Willoughby J, Harbison C, Fitzgerald K, Ilkayeva O, (2017). Divergent effects of glucose and fructose on hepatic lipogenesis and insulin signaling. J. Clin. Investig 127, 4059–4074. 10.1172/JCI94585. 28972537 PMC5663363

[14] Softic S, Cohen DE, and Kahn CR (2016). Role of Dietary Fructose and Hepatic De Novo Lipogenesis in Fatty Liver Disease. Dig. Dis. Sci 61, 1282–1293. 10.1007/s10620-016-4054-0. 26856717 PMC4838515

[15] Sugimoto K, Hosotani T, Kawasaki T, Nakagawa K, Hayashi S, Nakano Y, Inui H, and Yamanouchi T (2010). Eucalyptus leaf extract suppresses the postprandial elevation of portal, cardiac and peripheral fructose concentrations after sucrose ingestion in rats. J. Clin. Biochem. Nutr 46, 205–211. 10.3164/jcbn.09-93. 20490315 PMC2872225

[16] Jang C, Wada S, Yang S, Gosis B, Zeng X, Zhang Z, Shen Y, Lee G, Arany Z, and Rabinowitz JD (2020). The small intestine shields the liver from fructose-induced steatosis. Nat. Metab 2, 586–593. 10.1038/s42255-020-0222-9. 32694791 PMC8020332

[17] Chong MF-F, Fielding BA, and Frayn KN (2007). Mechanisms for the acute effect of fructose on postprandial lipemia. Am. J. Clin. Nutr 85, 1511–1520. 10.1093/ajcn/85.6.1511. 17556686

[18] Liu L, Li T, Liao Y, Wang Y, Gao Y, Hu H, Huang H, Wu F, Chen Y-G, Xu S, and Fu S (2020). Triose Kinase Controls the Lipogenic Potential of Fructose and Dietary Tolerance. Cell Metab. 32, 605–618.e7. 10.1016/j.cmet.2020.07.018. 32818435

[19] Jensen T, Abdelmalek MF, Sullivan S, Nadeau KJ, Green M, Roncal C, Nakagawa T, Kuwabara M, Sato Y, Kang DH, (2018). Fructose and sugar: A major mediator of non-alcoholic fatty liver disease. J. Hepatol 68, 1063–1075. 10.1016/j.jhep.2018.01.019. 29408694 PMC5893377

[20] Futatsugi K, Smith AC, Tu M, Raymer B, Ahn K, Coffey SB, Dowling MS, Fernando DP, Gutierrez JA, Huard K, (2020). Discovery of PF-06835919: A Potent Inhibitor of Ketohexokinase (KHK) for the Treatment of Metabolic Disorders Driven by the Overconsumption of Fructose. J. Med. Chem 63, 13546–13560. 10.1021/acs.jmedchem.0c00944. 32910646

[21] Shepherd EL, Saborano R, Northall E, Matsuda K, Ogino H, Yashiro H, Pickens J, Feaver RE, Cole BK, Hoang SA, (2021). Ketohexokinase inhibition improves NASH by reducing fructose-induced steatosis and fibrogenesis. JHEP Rep. 3, 100217. 10.1016/j.jhepr.2020.100217. 33490936 PMC7807164

[22] Karylowski O, Zeigerer A, Cohen A, and McGraw TE (2004). GLUT4 is retained by an intracellular cycle of vesicle formation and fusion with endosomes. Mol. Biol. Cell 15, 870–882. 10.1091/mbc.e03-07-0517. 14595108 PMC329400

[23] Garcia de Herreros A, and Birnbaum MJ (1989). The acquisition of increased insulin-responsive hexose transport in 3T3-L1 adipocytes correlates with expression of a novel transporter gene. J. Biol. Chem 264, 19994–19999. 2479643

[24] Mueckler M, and Thorens B (2013). The SLC2 (GLUT) family of membrane transporters. Mol. Aspects Med 34, 121–138. 10.1016/j.mam.2012.07.001. 23506862 PMC4104978

[25] Karim S, Adams DH, and Lalor PF (2012). Hepatic expression and cellular distribution of the glucose transporter family. World J. Gastroenterol 18, 6771–6781. 10.3748/wjg.v18.i46.6771. 23239915 PMC3520166

[26] Colville CA, Seatter MJ, Jess TJ, Gould GW, and Thomas HM (1993). Kinetic analysis of the liver-type (GLUT2) and brain-type (GLUT3) glucose transporters in Xenopus oocytes: substrate specificities and effects of transport inhibitors. Biochem. J 290, 701–706. 10.1042/bj2900701. 8457197 PMC1132337

[27] Wu L, Fritz JD, and Powers AC (1998). Different functional domains of GLUT2 glucose transporter are required for glucose affinity and substrate specificity. Endocrinology 139, 4205–4212. 10.1210/endo.139.10.6245. 9751501

[28] Guillam MT, Hümmler E, Schaerer E, Yeh JI, Birnbaum MJ, Beermann F, Schmidt A, Dériaz N, and Thorens B (1997). Early diabetes and abnormal postnatal pancreatic islet development in mice lacking Glut-2. Nat. Genet 17, 327–330. 10.1038/ng1197-327. 9354799

[29] Concha II, Velásquez FV, Martínez JM, Angulo C, Droppelmann A, Reyes AM, Slebe JC, Vera JC, and Golde DW (1997). Human erythrocytes express GLUT5 and transport fructose. Blood 89, 4190–4195. 10.1182/blood.v89.11.4190. 9166863

[30] Burant CF, Takeda J, Brot-Laroche E, Bell GI, and Davidson NO (1992). Fructose transporter in human spermatozoa and small intestine is GLUT5. J. Biol. Chem 267, 14523–14526. 1634504

[31] Buchs AE, Sasson S, Joost HG, and Cerasi E (1998). Characterization of GLUT5 Domains Responsible for Fructose Transport. Endocrinology 139, 827–831. 10.1210/endo.139.3.5780. 9492009

[32] Brown GK (2000). Glucose transporters: structure, function and consequences of deficiency. J. Inherit. Metab. Dis 23, 237–246. 10.1023/a:1005632012591. 10863940

[33] DeBosch BJ, Chen Z, Saben JL, Finck BN, and Moley KH (2014). Glucose transporter 8 (GLUT8) mediates fructose-induced de Novo lipogenesis and macrosteatosis. J. Biol. Chem 289, 10989–10998. 10.1074/jbc.M113.527002. 24519932 PMC4036240

[34] Ebert K, Ludwig M, Geillinger KE, Schoberth GC, Essenwanger J, Stolz J, Daniel H, and Witt H (2017). Reassessment of GLUT7 and GLUT9 as Putative Fructose and Glucose Transporters. J. Membr. Biol 250, 171–182. 10.1007/s00232-016-9945-7. 28083649

[35] Manolescu AR, Augustin R, Moley K, and Cheeseman C (2007). A highly conserved hydrophobic motif in the exofacial vestibule of fructose transporting SLC2A proteins acts as a critical determinant of their substrate selectivity. Mol. Membr. Biol 24, 455–463. 10.1080/09687680701298143. 17710649

[36] Cheeseman CI (1993). GLUT2 is the transporter for fructose across the rat intestinal basolateral membrane. Gastroenterology 105, 1050–1056. 10.1016/0016-5085(93)90948-c. 8405848

[37] Barone S, Fussell SL, Singh AK, Lucas F, Xu J, Kim C, Wu X, Yu Y, Amlal H, Seidler U, (2009). Slc2a5 (Glut5) is essential for the absorption of fructose in the intestine and generation of fructose-induced hypertension. J. Biol. Chem 284, 5056–5066. 10.1074/jbc.M808128200. 19091748 PMC2643499

[38] Conklin D, Gilbertson D, Taft DW, Maurer MF, Whitmore TE, Smith DL, Walker KM, Chen LH, Wattler S, Nehls M, and Lewis KB (1999). Identification of a Mammalian Angiopoietin-Related Protein Expressed Specifically in Liver. Genomics 62, 477–482. 10.1006/geno.1999.6041. 10644446

[39] Chang L, Chiang S-H, and Saltiel AR (2004). Insulin Signaling and the Regulation of Glucose Transport. Mol. Med 10, 65–71. 10.2119/2005-00029.Saltiel. 16307172 PMC1431367

[40] Boucher J, Kleinridders A, and Kahn CR (2014). Insulin receptor signaling in normal and insulin-resistant states. Cold Spring Harb. Perspect. Biol. 6, a009191. 10.1101/cshperspect.a009191. PMC3941218 24384568

[41] Koishi R, Ando Y, Ono M, Shimamura M, Yasumo H, Fujiwara T, Horikoshi H, and Furukawa H (2002). Angptl3 regulates lipid metabolism in mice. Nat. Genet 30, 151–157. 10.1038/ng814. 11788823

[42] Graham MJ, Lee RG, Brandt TA, Tai L-J, Fu W, Peralta R, Yu R, Hurh E, Paz E, McEvoy BW, (2017). Cardiovascular and Metabolic Effects of ANGPTL3 Antisense Oligonucleotides. N. Engl. J. Med 377, 222–232. 10.1056/NEJMoa1701329. 28538111

[43] Jung Y, Zhao M, and Svensson KJ (2020). Isolation, culture, and functional analysis of hepatocytes from mice with fatty liver disease. STAR Protoc. 1, 100222. 10.1016/j.xpro.2020.100222. 33377114 PMC7757664

[44] Coassolo L, Liu T, Jung Y, Taylor NP, Zhao M, Charville GW, Nissen SB, Yki-Jarvinen H, Altman RB, and Svensson KJ (2022). Mapping transcriptional heterogeneity and metabolic networks in fatty livers at single-cell resolution. iScience 26, 105802. 10.1016/j.isci.2022.105802. 36636354 PMC9830221

[45] Jang C, Hui S, Lu W, Cowan AJ, Morscher RJ, Lee G, Liu W, Tesz GJ, Birnbaum MJ, and Rabinowitz JD (2018). The Small Intestine Converts Dietary Fructose into Glucose and Organic Acids. Cell Metab. 27, 351–361.e3. 10.1016/j.cmet.2017.12.016. 29414685 PMC6032988

[46] Wood IS, and Trayhurn P (2003). Glucose transporters (GLUT and SGLT): expanded families of sugar transport proteins. Br. J. Nutr 89, 3–9. 10.1079/bjn2002763. 12568659

[47] Thorens B, and Mueckler M (2010). Glucose transporters in the 21st Century. Am. J. Physiol. Endocrinol. Metab 298, E141–E145. 10.1152/ajpendo.00712.2009. 20009031 PMC2822486

[48] DeBosch BJ, Chi M, and Moley KH (2012). Glucose transporter 8 (GLUT8) regulates enterocyte fructose transport and global mammalian fructose utilization. Endocrinology 153, 4181–4191. 10.1210/en.2012-1541. 22822162 PMC3423610

[49] Novelle MG, Bravo SB, Deshons M, Iglesias C, García-Vence M, Annells R, da Silva Lima N, Nogueiras R, Fernández-Rojo MA, Diéguez C, and Romero-Picó A (2021). Impact of liver-specific GLUT8 silencing on fructose-induced inflammation and omega oxidation. iScience 24, 102071. 10.1016/j.isci.2021.102071. 33554072 PMC7856473

[50] Membrez M, Hummler E, Beermann F, Haefliger J-A, Savioz R, Pedrazzini T, and Thorens B (2006). GLUT8 is dispensable for embryonic development but influences hippocampal neurogenesis and heart function. Mol. Cell Biol 26, 4268–4276. 10.1128/MCB.00081-06. 16705176 PMC1489108

[51] Teufel F, Almagro Armenteros JJ, Johansen AR, Gíslason MH, Pihl SI, Tsirigos KD, Winther O, Brunak S, von Heijne G, and Nielsen H (2022). SignalP 6.0 predicts all five types of signal peptides using protein language models. Nat. Biotechnol 40, 1023–1025. 10.1038/s41587-021-01156-3. 34980915 PMC9287161

[52] Yilmaz Y, Ulukaya E, Atug O, and Dolar E (2009). Serum concentrations of human angiopoietin-like protein 3 in patients with nonalcoholic fatty liver disease: association with insulin resistance. Eur. J. Gastroenterol. Hepatol 21, 1247–1251. 10.1097/MEG.0b013e32832b77ae. 19474742

[53] Butler AA, Graham JL, Stanhope KL, Wong S, King S, Bremer AA, Krauss RM, Hamilton J, and Havel PJ (2020). Role of angiopoietin-like protein 3 in sugar-induced dyslipidemia in rhesus macaques: suppression by fish oil or RNAi[S]. J. Lipid Res 61, 376–386. 10.1194/jlr.RA119000423. 31919051 PMC7053838

[54] Zhao S, Jang C, Liu J, Uehara K, Gilbert M, Izzo L, Zeng X, Trefely S, Fernandez S, Carrer A, (2020). Dietary fructose feeds hepatic lipogenesis via microbiota-derived acetate. Nature 579, 586–591. 10.1038/s41586-020-2101-7. 32214246 PMC7416516

[55] Petryszak R, Keays M, Tang YA, Fonseca NA, Barrera E, Burdett T, Füllgrabe A, Fuentes AMP, Jupp S, Koskinen S, (2016). Expression Atlas update - An integrated database of gene and protein expression in humans, animals and plants. Nucleic Acids Res. 44, D746–D752. 10.1093/nar/gkv1045. 26481351 PMC4702781

[56] Li X, Zhang Y, Zhang M, and Wang Y (2020). GALNT2 regulates ANGPTL3 cleavage in cells and in vivo of mice. Sci. Rep 10, 16168. 10.1038/s41598-020-73388-3. 32999434 PMC7527996

[57] Dewey FE, Gusarova V, Dunbar RL, O’Dushlaine C, Schurmann C, Gottesman O, McCarthy S, Van Hout CV, Bruse S, Dansky HM, (2017). Genetic and Pharmacologic Inactivation of ANGPTL3 and Cardiovascular Disease. N. Engl. J. Med 377, 211–221. 10.1056/NEJMoa1612790. 28538136 PMC5800308

[58] Shan L, Yu X-C, Liu Z, Hu Y, Sturgis LT, Miranda ML, and Liu Q (2009). The angiopoietin-like proteins ANGPTL3 and ANGPTL4 inhibit lipoprotein lipase activity through distinct mechanisms. J. Biol. Chem 284, 1419–1424. 10.1074/jbc.M808477200. 19028676 PMC3769808

[59] Tikka A, and Jauhiainen M (2016). The role of ANGPTL3 in controlling lipoprotein metabolism. Endocrine 52, 187–193. 10.1007/s12020-015-0838-9. 26754661 PMC4824806

[60] Haller JF, Mintah IJ, Shihanian LM, Stevis P, Buckler D, Alexa-Braun CA, Kleiner S, Banfi S, Cohen JC, Hobbs HH, (2017). ANGPTL8 requires ANGPTL3 to inhibit lipoprotein lipase and plasma triglyceride clearance. J. Lipid Res 58, 1166–1173. 10.1194/jlr.M075689. 28413163 PMC5454515

[61] Tarugi P, Bertolini S, and Calandra S (2019). Angiopoietin-like protein 3 (ANGPTL3) deficiency and familial combined hypolipidemia. J. Biomed. Res 33, 73–81. 10.7555/JBR.32.20170114. 29752428 PMC6477171

[62] Stanhope KL, Schwarz JM, Keim NL, Griffen SC, Bremer AA, Graham JL, Hatcher B, Cox CL, Dyachenko A, Zhang W, (2009). Consuming fructose-sweetened, not glucose-sweetened, beverages increases visceral adiposity and lipids and decreases insulin sensitivity in overweight/obese humans. J. Clin. Investig 119, 1322–1334. 10.1172/JCI37385. 19381015 PMC2673878

[63] Abdelmalek MF, Suzuki A, Guy C, Unalp-Arida A, Colvin R, Johnson RJ, and Diehl AM; Nonalcoholic Steatohepatitis Clinical Research Network (2010). Increased fructose consumption is associated with fibrosis severity in patients with nonalcoholic fatty liver disease. Hepatol. Baltim. Md 51, 1961–1971. 10.1002/hep.23535. PMC2922495 20301112

[64] Younossi ZM, Koenig AB, Abdelatif D, Fazel Y, Henry L, and Wymer M (2016). Global epidemiology of nonalcoholic fatty liver disease-Meta-analytic assessment of prevalence, incidence, and outcomes. Hepatol. Baltim. Md 64, 73–84. 10.1002/hep.28431. 26707365

[65] Douard V, Choi H-I, Elshenawy S, Lagunoff D, and Ferraris RP (2008). Developmental reprogramming of rat GLUT5 requires glucocorticoid receptor translocation to the nucleus. J. Physiol 586, 3657–3673. 10.1113/jphysiol.2008.155226. 18556366 PMC2538831

[66] Dotimas JR, Lee AW, Schmider AB, Carroll SH, Shah A, Bilen J, Elliott KR, Myers RB, Soberman RJ, Yoshioka J, and Lee RT (2016). Diabetes regulates fructose absorption through thioredoxin-interacting protein. eLife 5, e18313. 10.7554/elife.18313. 27725089 PMC5059142

[67] Karim S, Liaskou E, Fear J, Garg A, Reynolds G, Claridge L, Adams DH, Newsome PN, and Lalor PF (2014). Dysregulated hepatic expression of glucose transporters in chronic disease: contribution of semicarbazide-sensitive amine oxidase to hepatic glucose uptake. Am. J. Physiol. Gastrointest. Liver Physiol 307, G1180–G1190. 10.1152/ajpgi.00377.2013. 25342050 PMC4269679

[68] Carayannopoulos MO, Chi MM, Cui Y, Pingsterhaus JM, McKnight RA, Mueckler M, Devaskar SU, and Moley KH (2000). GLUT8 is a glucose transporter responsible for insulin-stimulated glucose uptake in the blastocyst. Proc. Natl. Acad. Sci. USA 97, 7313–7318. 10.1073/pnas.97.13.7313. 10860996 PMC16542

[69] Pinto AB, Carayannopoulos MO, Hoehn A, Dowd L, and Moley KH (2002). Glucose transporter 8 expression and translocation are critical for murine blastocyst survival. Biol. Reprod 66, 1729–1733. 10.1095/biolreprod66.6.1729. 12021054

[70] Zhang CC, Kaba M, Ge G, Xie K, Tong W, Hug C, and Lodish HF (2006). Angiopoietin-like proteins stimulate ex vivo expansion of hematopoietic stem cells. Nat. Med 12, 240–245. 10.1038/nm1342. 16429146 PMC2771412

[71] Camenisch G, Pisabarro MT, Sherman D, Kowalski J, Nagel M, Hass P, Xie M-H, Gurney A, Bodary S, Liang XH, (2002). ANGPTL3 stimulates endothelial cell adhesion and migration via integrin alpha vbeta 3 and induces blood vessel formation in vivo. J. Biol. Chem 277, 17281–17290. 10.1074/jbc.M109768200. 11877390

[72] Kersten S. (2017). Angiopoietin-like 3 in lipoprotein metabolism. Nat. Rev. Endocrinol 13, 731–739. 10.1038/nrendo.2017.119. 28984319

[73] Tang J, Ma S, Gao Y, Zeng F, Feng Y, Guo C, Hu L, Yang L, Chen Y, Zhang Q, (2022). ANGPTL8 promotes adipogenic differentiation of mesenchymal stem cells: potential role in ectopic lipid deposition. Front Endocrinol (Lausanne) 13, 927763. 10.3389/fendo.2022.927763. 36034432 PMC9404696

[74] Jiang Z, Zhao M, Voilquin L, Jung Y, Aikio MA, Sahai T, Dou FY, Roche AM, Carcamo-Orive I, Knowles JW, (2021). Isthmin-1 is an adipokine that promotes glucose uptake and improves glucose tolerance and hepatic steatosis. Cell Metab 33, 1836–1852.e11. 10.1016/j.cmet.2021.07.010. 34348115 PMC8429235

[75] Radhakrishnan S, Ke JY, and Pellizzon MA (2020). Targeted Nutrient Modifications in Purified Diets Differentially Affect Nonalcoholic Fatty Liver Disease and Metabolic Disease Development in Rodent Models. Curr. Dev. Nutr 4, nzaa078. 10.1093/cdn/nzaa078. 32494762 PMC7250583

