# Supplementary material for: ANGPTL3 orchestrates hepatic fructose sensing and metabolism
Source: Cell Rep. Author manuscript; Available in PMC 2025 Aug 5. (PMC12324827; doi:10.1016/j.celrep.2025.115962)
Supplement: 1 [file NIHMS2099522-supplement-1.pdf]

Cell Reports, Volume *44*

## Supplemental information

# ANGPTL3 orchestrates hepatic fructose sensing and metabolism

**Meng Zhao, Karen Y. Linde-Garelli, Zeyuan Zhang, David Toomer, Saranya C. Reghupaty, John Isaiah Jimenez, Laetitia Coassolo, Lianna W. Wat, Daniel Fernandez, and Katrin J. Svensson**

## Figure S1

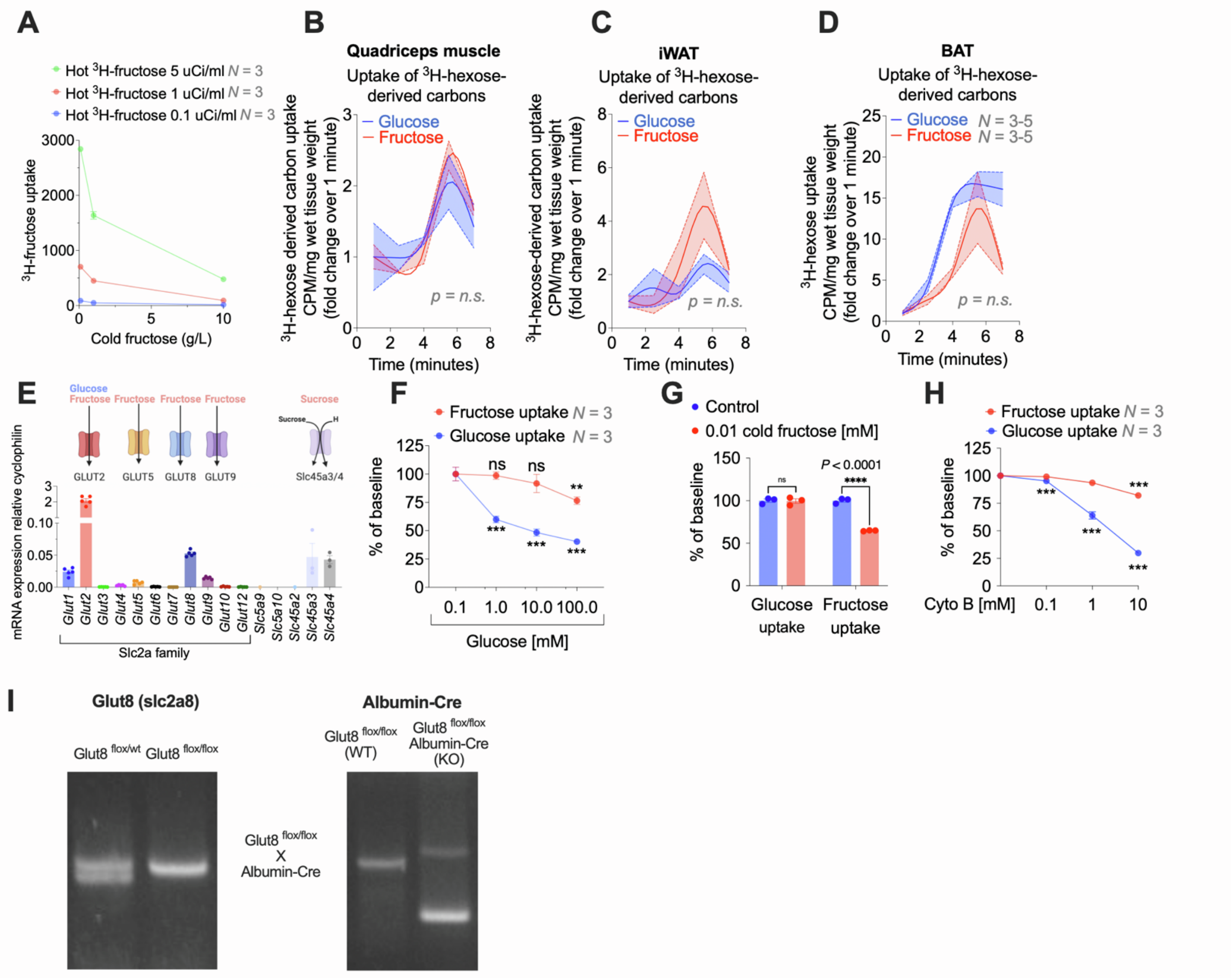


**Figure S1, Related to Figure 1. Validation of *in vitro* and *in vivo* uptake of fructose and fructose-derived carbons.**

A. Fructose uptake measured by [3H]-fructose incorporation in AML12 cells at indicated doses of hot [3H]-fructose and cold (unlabeled) fructose ($N$ = 3 samples per group).

B. Time-dependent *in vivo* uptake of fructose-or glucose-derived carbons in quadriceps skeletal muscle measured by [3H]-fructose or [3H]-glucose incorporation in male mice at 1, 2.5, 4, 5.5, and 7 minutes post I.P. injection ($N$ = 3-5 mice per group).

C. Time-dependent *in vivo* uptake of fructose-or glucose-derived carbons in inguinal white fat (iWAT) measured by [3H]-fructose or [3H]-glucose incorporation in male mice at 1, 2.5, 4, 5.5, and 7 minutes post I.P. injection ($N$ = 3-5 mice per group).

D. Time-dependent *in vivo* uptake of fructose-or glucose-derived carbons in brown fat (BAT) measured by [3H]-fructose or [3H]-glucose incorporation in male mice at 1, 2.5, 4, 5.5, and 7 minutes post I.P. injection ($N$ = 3-5 mice per group).

E. Relative gene expression analysis of hexose transporters in male mouse livers ($N$ = 5 mice per group).

F. Fructose or glucose uptake measured by [3H]-fructose or [3H]-glucose incorporation with increasing levels of glucose treatments in HepG2 cells ($N$ = 3 samples per group).

G. Fructose or glucose uptake measured by [3H]-fructose or [3H]-glucose incorporation with additional fructose treatments in HepG2 cells ($N$ = 3 samples per group).

H. Comparison of fructose and glucose uptake measured by [3H]-fructose or [3H]-glucose incorporation in AML12 cells treated with indicated doses of CytoB for 5 minutes ($N$ = 3 samples per group).

I. Representative genotyping of liver-specific male Glut8-KO mice ($N$ = 1 mouse per genotype)

Data are presented as mean ± SEM. *p < 0.05, **p < 0.01, ***p < 0.001 by two-way ANOVA (F, G, H).

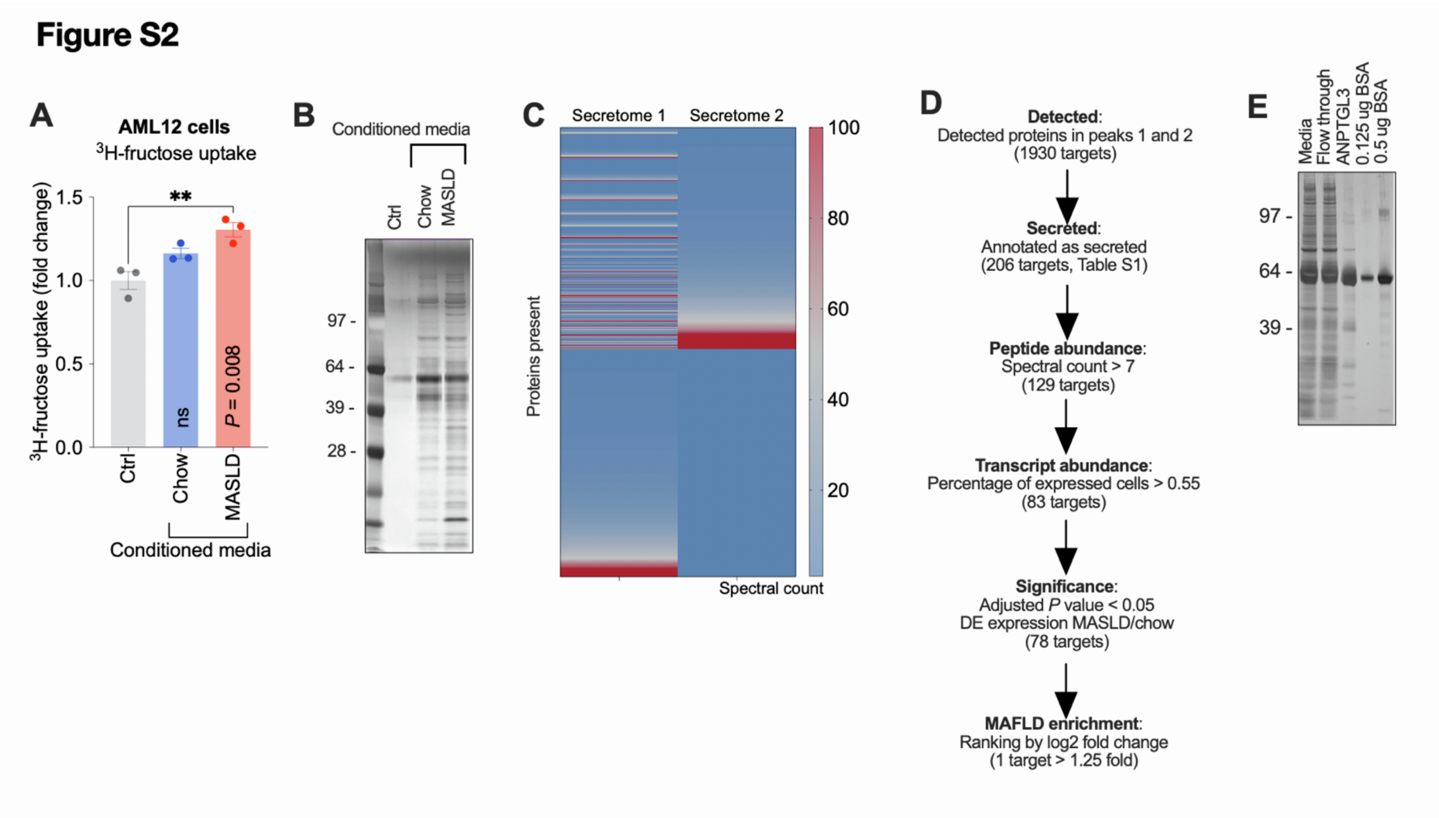


**Figure S2, Related to Figure 2. Identification of Angptl3 from the mouse hepatocyte secretome.**

A. Fructose uptake measured by [3H]-fructose incorporation in AML12 cells treated with conditioned medium from chow or MASLD hepatocytes (*N* = 3 samples per group, repeated in three independent experiments).

B. Representative silver stain of conditioned media from primary mouse hepatocytes isolated from male chow and MASLD mice after 24 hours (repeated in three independent experiments).

C. Heatmap of proteins identified from Secretome 1 and Secretome 2 by unbiased proteomics. Protein intensities in spectral count are displayed as colors ranging from blue to red as shown in the color scale.

D. Filtering criteria to identify MASH-induced hepatokines that are enriched in conditioned media fractions that induce hepatocyte fructose uptake.

E. Representative silver stain of recombinant mouse Angptl3 (repeated in three independent experiments).

Data are presented as mean ± SEM. $*p < 0.05$, $**p < 0.01$, $***p < 0.001$ by one-way ANOVA (A).

## Figure S3

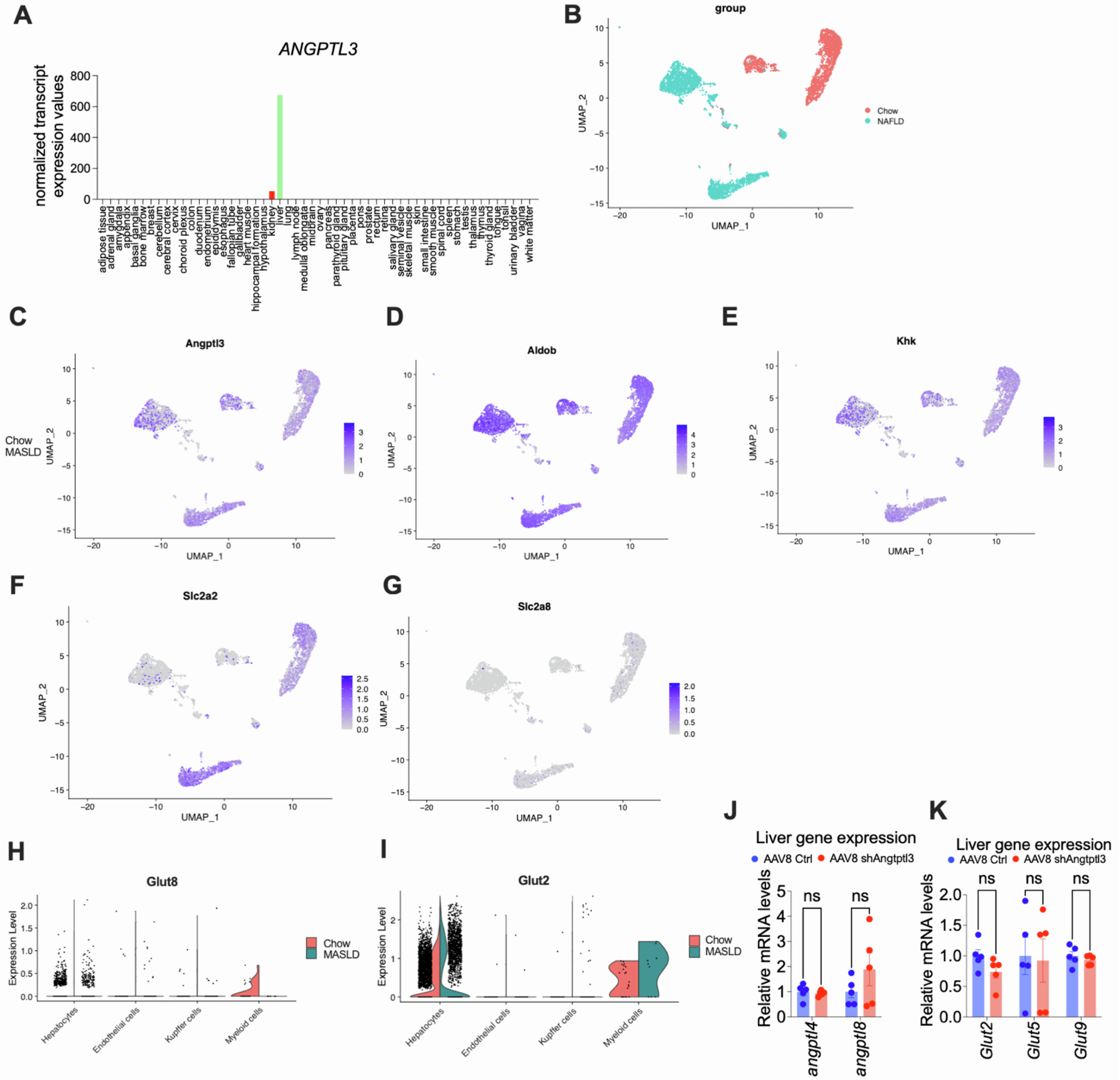


**Figure S3, Related to Figure 3. Characterization and expression of hexose transporters and fructolysis genes in mice.**

Gene expression of ANGPTL3 in human organs. Data from Human Protein Atlas.

A. UMAP of cell types separated by chow and MASLD (*N* = 5 male mice per group).

C-G. UMAP of hepatocyte-enriched genes *angptl3, khk (khk-a and khk-c), aldob, slc2a2,* and *slc2a8* across all cell clusters. (*N* = 5 male mice per group).

H-I. Gene expression levels of *Glut2,* and *Glut8* in male chow and MASLD mouse livers measured by single-cell RNA sequencing (*N* = 5 mice per group).

J. Relative gene expression levels of *angptl4* and *angptl8* in livers from AAV8-Ctl and AAV8-shAngptl3 treated male mice (*N* = 5 mice per group).

K. Relative gene expression levels of *glut2, glut5,* and *glut9* in livers from AAV8-Ctl and AAV8-shAngptl3 treated male mice (*N* = 5 mice per group).

Data are presented as mean ± SEM. *p < 0.05, **p < 0.01, ***p < 0.001 by two-way ANOVA (J, K).

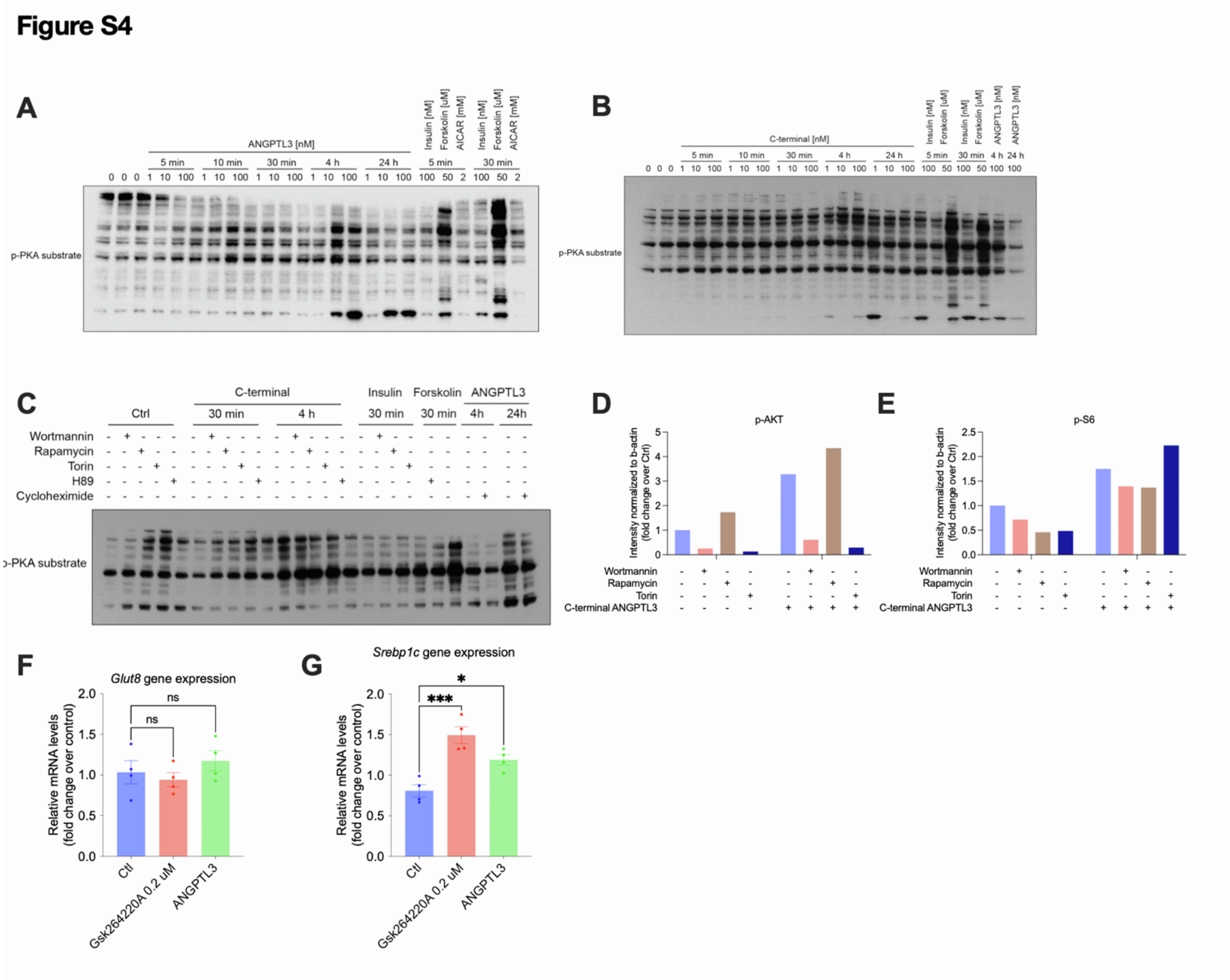

**Figure S4, Related to Figure 4. Gene expression and signaling functions of ANGPTL3.**

A. Representative western blot of p-PKA in HepG2 cells treated with indicated doses of full-length human ANGPTL3 and positive controls showing the intracellular signaling pathways over time (*N* = 1 sample per group, repeated in two independent experiments).

B. Representative western blot of p-PKA in HepG2 cells treated with indicated doses of C-terminal human ANGPTL3 and positive controls showing the intracellular signaling pathways over time (*N* = 1 sample per group, repeated in two independent experiments).

C. Representative western blot of p-PKA signaling induced by C-terminal human ANGPTL3 (100 nM) or positive controls in HepG2 cells pre-treated with PI3K inhibitors Wortmannin (1 μM), mTORC1 inhibitor rapamycin (100 nM), mTORC1/2 dual inhibitor torin (100 nM), PKA inhibitor H89 (10 μM), or the translation inhibitor cycloheximide (10 μM).

D-E. Quantification of p-AKT and p-S6 induced by C-terminal human ANGPTL3 (100 nM) or positive controls in HepG2 cells pre-treated with PI3K inhibitors Wortmannin (1 μM), mTORC1 inhibitor rapamycin (100 nM), or mTORC1/2 dual inhibitor torin (100 nM) (*N* = 1 sample per group).

F. Relative gene expression levels of *Glut8* in AML12 cells treated with Gsk264220A or ANGPTL3 (*N* = 4 samples per group).

G. Relative gene expression levels of *Srebp1c* in AML12 cells treated with Gsk264220A or ANGPTL3 (*N* = 4 samples per group).

Data are presented as mean ± SEM. *$p < 0.05$, **$p < 0.01$, ***$p < 0.001$ by one-way ANOVA (F, G).
